# *Cotinus coggygria* Scop. extract-loaded polycaprolactone/hyaluronic acid nanofibers as candidate wound dressings: *in vitro* characterization, cytocompatibility and antibacterial evaluation

**DOI:** 10.3389/fphar.2026.1948812

**Published:** 2026-10-23

**Authors:** Eylul Su Saral-Acarca, Serdar Tort, Mustafa Türk, Fatma Sezer Senol Deniz, Ilkay Erdogan Orhan, Sibel Ilbasmis-Tamer

**Affiliations:** 1 Department of Pharmaceutical Technology, Faculty of Pharmacy, Gazi University, Ankara, Türkiye; 2 Department of Pharmaceutical Technology, Faculty of Pharmacy, Başkent University, Ankara, Türkiye; 3 Department of Bioengineering, Faculty of Engineering, Kırıkkale University, Kırıkkale, Türkiye; 4 Department of Pharmacognosy, Faculty of Pharmacy, Gazi University, Ankara, Türkiye; 5 Department of Pharmacognosy, Faculty of Pharmacy, Lokman Hekim University, Ankara, Türkiye

**Keywords:** *Cotinus coggygria*, nanofiber, scratch, wound dressing, wound healing

## Abstract

*Cotinus coggygria* Scop. (Anacardiaceae) possesses antibacterial, antioxidant, anticancer, and wound-healing properties. Hyaluronic acid (HA), a key extracellular matrix component, supports tissue regeneration. In this study, electrospun polycaprolactone (PCL) nanofibers containing *C. coggygria* extract and HA were developed as wound dressings. Optimized polymer solutions and electrospinning parameters were used according to material composition. Nanofibers were characterized by contact angle measurements, texture analysis, scanning electron microscopy, and *in vitro* cell culture assays. The fiber diameters ranged from 300 to 700 nm, and contact angle decreased to 65.3°, indicating improved hydrophilicity due to HA and *C*. *coggygria* extract incorporation. The addition of extract and HA yielded a maximum tensile strength of 2.40 MPa. After 24 h of exposure, the F5 maintained L929 fibroblast viability above 80% across the entire tested range (0.062–1 mg/mL). At 48 h, F5 produced the lowest apoptotic (6% ± 1%) and necrotic (10% ± 1%) indices among the extract-containing fibers at 1 mg/mL, indicating cytocompatibility towards L929 fibroblasts under the conditions tested. Antibacterial tests revealed inhibition zones of 21 ± 0 mm against *Staphylococcus aureus* and 6 ± 1 mm against *Escherichia coli*. Overall, PCL nanofibers incorporating *C. coggygria* extract and HA demonstrate promising potential for wound healing applications owing to their favorable mechanical properties, high biocompatibility, and notable antibacterial activity.

## 1 Introduction

Novel pharmaceutical approaches have been introduced for wound therapy. Semi-solid dosage forms such as cream and paste are formulations that are easy to use but novel systems provide many advantages. Wound dressings have an important role in wound healing therapy as they prevent the wound surface from coming into contact with contamination factors and protect the treatment process from external factors. Nanofibers are very suitable systems to be used as wound dressings. These systems have fibers ranging typically from 100 to 1,000 nm in diameter. Because of their high surface-area-to-volume ratio and the capacity to carry bioactive compounds, accelerating the healing process can be achieved effectively. (Inal and Mulazimoglu, 2019; Uppal et al., 2011). Electrospun fiber production can be adjusted into a wide range of fiber morphologies, and the electrospinning method is a highly adaptable technology. These electrospun nanofibers have a variety of morphological shapes, including core shells, porous structures, aligned or randomly oriented nanofiber diameters, and more, employing a single or mixed copolymer and optionally adding additional active ingredients (Fadil et al., 2021). It is also possible to load various medications, such as antibiotics and anticancer drug substances, as well as biomacromolecules into nanofibers. Nanofiber applications for biosensors, tissue engineering, wound dressings, and medication delivery systems are also available (Ilbasmis-Tamer et al., 2022).

Electrospinning is a versatile and widely adopted technique in skin tissue engineering due to its ability to fabricate nanofibrous scaffolds that closely mimic the native extracellular matrix (ECM). Furthermore, the wide range of natural and synthetic polymers that can be electrospun allows for the customization of scaffold properties to match specific tissue requirements. These scaffolds can also be engineered to enhancing wound healing processes, while the relative simplicity and cost-effectiveness of the process make it suitable for large-scale production. Despite these advantages, electrospinning has certain limitations, including the use of organic solvents that can be cytotoxic and necessitate thorough post-processing to ensure biocompatibility. Additionally, electrospun nanofibrous scaffolds often exhibit limited mechanical strength, which may not be sufficient for load-bearing applications without additional reinforcement, and achieving uniform fiber alignment remains challenging yet crucial for directing cell growth and function in tissue engineering applications. Nanofiber production *via* electrospinning begins with the polymer solution, held in a syringe connected to a constant flow pump, being delivered through an inert line to the tip of a nozzle with a specified diameter or configuration (e.g., core/shell). Furthermore, the electrospinning method is effective for encapsulating bioactive substances, which are essential for the management of long-term wounds (Khan et al., 2021).

Polycaprolactone (PCL) is a biodegradable and biocompatible synthetic polymer widely used in biomedical applications, particularly where mechanical durability is desired. Furthermore, compared to natural biopolymers such as chitosan, gelatin, and collagen, PCL provides greater mechanical strength, slower degradation, and superior processability for electrospinning nanofibers. However, its hydrophobic nature and lack of inherent bioactivity can limit cell adhesion and proliferation unless modified or combined with bioactive agents (Lababidi et al., 2025). To address this, hyaluronic acid (HA) can be incorporated, offering superior biological activity, particularly in cell signalling and hydration, which makes it highly effective in promoting tissue regeneration (Fahimirad et al., 2021; Rahman et al., 2024).

The human body naturally contains HA, which supports the extracellular matrix (ECM) by binding and retaining water, facilitating wound healing, and mediating cellular signalling. Acting as a transient scaffolding, HA permits the diffusion of nutrients and waste products during the wound-healing process while also influencing the migration and proliferation of keratinocytes (Astaneh and Fereydouni, 2023). Its strong hydrophilicity and high biocompatibility, combined with its ability to modulate inflammation and angiogenesis, make it particularly effective in promoting wound healing. However, when used alone, HA suffers from rapid degradation and limited mechanical strength, which compromises its structural stability in wound dressing applications (Huerta-Ángeles and Mixcoha, 2025).

The combination of PCL and HA was intentionally chosen to harness their complementary properties, where PCL serves as a structural backbone providing durability and a platform for the sustained delivery of bioactive compounds, while HA enhances biocompatibility, supports cellular activity, and boosts the wound-healing efficacy of the *C. coggygria* extract. Integrating PCL and HA allows the resulting nanofiber matrix to overcome the individual limitations of each polymer, creating a synergy that supports both mechanical integrity and the biological interactions essential for effective wound healing.

*C. coggygria* is the sole species of its genus grown in Türkiye, where it exhibits a wide distribution across Southern Europe and Anatolia. Traditionally, the roots of the plant, sometimes referred to as “yellow root or yellow wood”, were employed to dye yarn, leather, and fabric with a yellow hue, while in Anatolian folk medicine, the leaves have been ingested as a 5% infusion for their antibacterial, astringent, hemostatic, and antipyretic properties. Furthermore, there exists documentation of its utilization as a therapeutic agent for treating wounds both domestically and in Russia (Mamedov et al., 2005; Kultur, 2007; Baytop, 1999). Pharmacological research has established that *C. coggygria* exhibits significant antioxidant, anticancer, antigenotoxic, antimicrobial, antiviral, hepatoprotective, anti-inflammatory, antiulcer, and wound-healing properties, which are largely attributed to its major secondary metabolites, including flavonoids, anthocyanins, tannins, and other phenolic compounds. (Sen et al., 2023; Matic et al., 2016). In our previous study, flavonoids such as astragalin, hyperoside, isoquercetin, and the phenolic compound methyl gallate were isolated from the pedicel extract of the plant using an activity-guided fractionation process targeting the inhibition of elastase and collagenase associated with inflammation and wound pathologies (Deniz et al., 2020). These findings led to the acquisition of *C. coggygria* dry extract from Vemo® (Bulgaria) for subsequent investigations. Traditionally, *C. coggygria* leaves have been widely utilized in folk medicine for their antipyretic, antiseptic, antihemorrhagic, anti-diarrheal, and wound-healing effects (Aksoy et al., 2016), with wound healing being the most frequently cited benefit across diverse regional traditions. Furthermore, scientific studies have demonstrated that the antibacterial activity of *C. coggygria* extracts and essential oils directly supports their traditional wound-healing efficacy. Additionally, studies have demonstrated that the antibacterial activity of the wound-healing action supports the *C. coggygria* extract and oils (Antal et al., 2021). The extract is expected to contribute two properties that PCL does not possess: antibacterial activity against the Gram-positive organisms that dominate the early wound flora, attributable to its flavonoid and tannin content, and hydrophilicity arising from its water-soluble constituents. Both expectations are tested directly in this work, and both are shown to be concentration-dependent.

Although *C. coggygria* has been repeatedly shown to promote wound healing *in vivo*, in every one of these reports the extract was applied as a free solution, ointment or gel (Aksoy et al., 2016; Bozkaya et al., 2023); it has not been incorporated into a fibrous matrix, and it is not known whether its activity survives the electrospinning process or whether it can be delivered from a solid dressing. Conversely, the extract-loaded electrospun PCL dressings reported so far have combined the polymer with either a second structural polymer or a nanoparticulate carrier and have not combined a hydrophilic extracellular-matrix component with a standardized polyphenol-rich plant extract within the same fiber (Table 1). The present study therefore addresses two specific questions: (i) whether a standardized *C. coggygria* leaf extract can be electrospun into a PCL matrix while retaining fiber morphology, mechanical integrity and antibacterial activity; and (ii) whether the addition of hyaluronic acid can offset the hydrophobicity of PCL and the concentration-dependent cytotoxicity of the extract. The study is designed as an *in vitro* formulation-characterization study, and no claim is made beyond the endpoints measured here.

**TABLE 1:** Comparison of different polymer systems and activities.

Polymer system	Bioactive incorporated	Reported *in vitro*/*in vivo* outcome	References
Gelatin	Silver-based bactericidal agent	Antibacterial nanofibrous dressing	(Inal and Mulazimoglu, 2019)
Hyaluronic acid	- (polymer only)	Improved healing vs. gauze/vaseline *in vivo*	(Uppal et al., 2011)
PCL/chitosan	Curcumin-loaded chitosan nanoparticles	Cytocompatibility, antibacterial, *in vivo* healing	(Fahimirad et al., 2021)
PCL/collagen	Acanthophora spicifera extract-mediated CuO NPs	Biocompatibility, anti-hemolytic, antibacterial	(Sivakumar et al., 2024)
PCL	Jaft (Quercus sp.) extract	Histological comparison of healing quality	(Samavati et al., 2023)
PCL/gelatin	Saussurea costus extract	Wound-healing activity	(Lababidi et al., 2025)
PCL	Halloysite nanotubes	Antibacterial, mechanical reinforcement	(Khunova et al., 2022)
Chitosan/PEO	- (polymer only)	Anti-adhesive, antibacterial, breathable	(Jia et al., 2024)
- (free extract, no dressing)	C. coggygria ethanol extract	Accelerated healing in diabetic rats	(Aksoy et al., 2016)
- (free extract, no dressing)	C. coggygria extract	Biocompatibility and *in vivo* healing	(Bozkaya et al., 2023)
PCL/HA (this work)	C. coggygria standardized leaf extract (1%–3%)	*In vitro* characterization, cytocompatibility, scratch closure, antibacterial	Present study

## 2 Materials and methods

### 2.1 Materials

Commercial *C. coggygria* dry standardized extract supplied from Vemo® (VEMO Corp., Sofia, Bulgaria). According to the manufacturer’s specification sheet, *C*. *coggygria* leaf extract contains not less than 30.0% total polyphenols (calculated as catechin), 15.0% flavonoids (calculated as apigenin), 2.0% flavonoids (calculated as quercetin), and 32.0% tannic compounds (calculated as tannin) on a dry substance basis (available at https://vemocorp.com/products/herbal-extracts/cotinus-coggygria/). PCL (80 kDa) and HA (400 kDa) were procured from Sigma-Aldrich (Merck KGaA, Germany) and Kadioglu Medikal (Ankara, Türkiye), respectively, while analytical-grade dimethylformamide (DMF), acetone, and dimethyl sulfoxide (DMSO) were purchased from Sigma-Aldrich (Merck KGaA, Germany). These specific molecular weights were deliberately selected to optimize electrospinning performance and biomedical utility. Specifically, PCL at 80 kDa provides an ideal balance between mechanical strength and biodegradability for scaffold fabrication, whereas HA at 400 kDa imparts favorable viscoelastic properties and biological activity critical for wound-healing applications. Dulbecco’s modified Eagle’s medium (DMEM), fetal bovine serum (FBS), penicillin/streptomycin, and trypsin-ethylenediaminetetraacetic acid (EDTA) (Biological Industries and Sartorius, Göttingen, Germany); 3-(4,5-dimethylthiazol-2-yl)-2,5-diphenyltetrazolium bromide (MTT), isopropanol, DMSO, phosphate-buffered saline (PBS, Sigma-Aldrich, Merck KGaA, Germany); ribonuclease A, propidium iodide, Hoechst 33,342 (Serva, Heidelberg, Germany); nutrient broth, Mueller–Hinton agar, erythromycin 15 µg disks, blank paper disks, McFarland standards (Sigma-Aldrich, Merck KGaA, Germany); culture plasticware (Corning Incorporated, New York, United States of America); Folin–Ciocalteu reagent, gallic acid, quercetin, aluminium chloride (Sigma-Aldrich, Merck KGaA, Germany).

### 2.2 Production of electrospun nanofibers

Prior to electrospinning, six distinct formulations were prepared using tailored solvent systems selected on the basis of literature data and the solubility requirements of PCL and *Cotinus* extract. Specifically, an acetone-DMF (5:5, v/v) solvent system was employed for HA-free formulations (F1, F3, and F4), whereas an acetone-DMF-water (5:4:1, v/v/v) system was utilized for HA-containing formulations (F2, F5, and F6) to facilitate the controlled hydration and swelling of HA. Water was required in the HA-containing solutions because HA is not soluble in acetone/DMF and does not hydrate without it. All concentrations are expressed as % (w/v) with respect to the total volume of the solvent system. Thus, a 10% PCL/0.5% HA/1% extract solution contained 1.00 g PCL, 50 mg HA and 100 mg extract *per* 10 mL of solvent. To prevent any potential phase separation, the mixtures were thoroughly blended immediately prior to electrospinning, and the process duration was kept strictly controlled.

To achieve uniform and bead-free nanofibers, preliminary experiments were conducted to systematically optimize the electrospinning parameters based on resulting fiber morphology and consistency. Electrospinning was carried out on a single nozzle electrospinning unit (Inovenso NE300, Istanbul, Türkiye) with a drum collector. All fabrication was performed at room conditions. The optimal operational conditions were determined to be an applied voltage of 23 kV, a flow rate ranging from 0.8 to 1.0 mL/h, and a needle-to-collector distance of 10–12 cm. The extract-containing solutions required a lower flow rate and a longer working distance because at 1.0 mL/h and 10 cm they produced an unstable jet with wet deposition, the extract having increased the solution conductivity. These fine-tuned parameters ensured the successful fabrication of continuous, uniform nanofibers well-suited for advanced wound-dressing applications. Process parameters and compositions of polymer solutions are given in Table 2. Initially, PCL was dissolved in the solvent system and stirred until a clear solution was formed, followed by the sequential addition of HA and *C. coggygria* extract. Once a homogeneous mixture was achieved, the polymer solution was transferred into a syringe, loaded onto a syringe pump, and electrospun continuously. Notably, the production of extract-containing formulations required a reduced feed rate and an increased needle-to-collector distance. Each formulation was electrospun continuously for 5–6 h onto a nonwoven backing fabric. The resulting nanofibers were collected on a nonwoven fabric backing and stored at low temperatures until further characterization, after which the optimal formulations were determined.

**TABLE 2:** Compositions of polymer solutions and production parameters.

Formulation code	Polycaprolactone (%)	Hyaluronic acid (%)	Cotinus coggygria extract (%)	Process parameters (distance, voltage, flow rate)
F1	10	-	-	10 cm, 23 kV, 1 mL/h
F2	10	0.5	-	10 cm, 23 kV, 1 mL/h
F3	10	-	1	12 cm, 23 kV, 0.8 mL/h
F4	10	-	3	12 cm, 23 kV, 0.8 mL/h
F5	10	0.5	1	12 cm, 23 kV, 0.8 mL/h
F6	10	0.5	3	12 cm, 23 kV, 0.8 mL/h

### 2.3 Characterizations of electrospun nanofibers

#### 2.3.1 Scanning electron microscopy measurements

The morphology and diameter distribution of the nanofibers were examined using scanning electron microscope (SEM) (JEOL JSM-6060, Tokyo, Japan). Micrographs were analyzed with ImageJ software (National Institutes of Health, Bethesda, MD, United States of America), and fiber diameters were measured on SEM fields from a single fabrication batch per formulation, with 50 fibers measured *per* formulation. These 50 values are technical replicates from one batch and do not constitute independent experimental units. Fiber diameters are therefore reported as descriptive statistics (mean ± SD of the measured population) and are not subjected to inferential testing between formulations. The batch-to-batch reproducibility of fiber diameter was not assessed, and this is stated as a limitation.

#### 2.3.2 Contact angle study

Wettability is a critical property for wound dressings, as it facilitates the absorption of excess exudates and maintains a moist wound healing environment. Although PCL is inherently hydrophobic, the incorporation of HA enhances surface wettability. To evaluate these surface characteristics, contact angle measurements were performed using an Attension Theta Lite optical tensiometer (Biolin Scientific AB, Gothenburg, Sweden) *via* the sessile drop technique. Specifically, a 5 μL droplet of distilled water was placed onto the nanofibrous scaffold mounted on the sample holder to determine the contact angle values (Tort et al., 2017). Three measurements were carried out.

#### 2.3.3 Mechanical properties

Mechanical properties such as flexibility or strength of wound dressings are vital for healing therapy. Tensile testing was performed on a TA. XTplus texture analyser (Stable Micro Systems, Godalming, United Kingdom) fitted with a tensile grip, at 23 °C ± 2 °C. Rectangular specimens measuring 10 × 30 mm were cut from the fibers. Three measurements were carried out.

#### 2.3.4 DSC, FT-IR and Confocal Raman spectroscopy analysis

Differential scanning calorimetry (DSC, Shimadzu, DSC-60, Japan) examination was conducted on *C. coggygria* extract, PCL, HA, and F5 formulation. The temperature range for the samples was 25 °C–300 °C. Fourier transform infrared (FT-IR) spectroscopy (Thermo Nicolet Avatar 370 spectrometer, United States of America) was used to analyze *C*. *coggygria* extract, PCL, HA, and F5 formulation in transmission mode within the wave number range of 650–4,000 cm^−1^. Confocal Raman spectroscopy (NRS4500, JASCO Corporation, Hachioji, Tokyo, Japan) with a 785-nm laser source and charge-coupled device (CCD) detector was used to detect the characteristic peaks of the F5 formulation and the substances in the formulation. A MPLN ×10 objective was used for measurements.

### 2.4 Cell culture studies

#### 2.4.1 Materials for cell culture

L929 mouse fibroblasts were obtained from the cell culture stocks of the Kırıkkale University Scientific and Technological Research Application and Research Center (KÜBTUAM, Ankara, Türkiye). (Note: They were purchased by the laboratory from the Foot-and-Mouth Disease Institute) The cells were maintained in DMEM supplemented with 10% FBS and 1% penicillin/streptomycin. For subculturing, cells were detached using Trypsin-EDTA (Biological Industries and Sartorius, Göttingen, Germany) and washed with phosphate-buffered saline (PBS), with cell viability and counting assessed via trypan blue exclusion. Apoptosis and necrosis rates were determined using a double-staining protocol with Ribonuclease-A, propidium iodide (Serva, Heidelberg, Germany), and Hoechst 33,342 (Serva, Heidelberg, Germany). All *in vitro* culture assays were performed using standard culture dishes and multi-well plates (Corning, Incorporated, New York, United States of America).

#### 2.4.2 Cell culture

Frozen L929 cells were rapidly thawed in a 37 °C water bath. Under a sterile laminar flow cabinet, the cell suspension was transferred to a 15 mL Falcon tube and centrifuged at 2000 rpm for 2 min. The resulting cell pellet was resuspended in 3 mL of complete DMEM medium (supplemented with 10% FBS and 1% antibiotics), gently homogenized, and seeded into a 75 cm^2^ culture flask. The cultures were then maintained in a humidified incubator at 37 °C with an atmosphere of 5% CO_2_.

#### 2.4.3 *In vitro* cell viability assay

MTT test was performed to determine cell viability. The study was conducted according to the ISO 10993-5/MTT cytotoxicity test protocol. The samples were sterilized by exposure to UV light in a laminar flow cabinet for 10 min. Cells were removed from the flasks enzymatically (trypsin/EDTA, 0.05%) and the cell suspension was centrifuged (2000 rpm 2 min). Then, the cells were resuspended in the culture medium and planted in a 96-well cell culture dish at a cell density of 1 × 10^4^ cells/mL. The transplanted cells were incubated for 24 h to ensure 80% confluence. Extraction was performed by incubating the sterilized material in serum-containing medium (at a concentration of 1 mg/mL) at 37 °C for 24 h, and 100 µL of this extract was added via serial dilution (The extract obtained in this manner was also used for the apoptosis/necrosis assay). After 24 h of incubation, the cells in the culture dishes were examined with a microscope, the culture medium in each well was carefully withdrawn, 50 µL of MTT (1 mg/mL) solution was added to each well, and the dishes were incubated in the incubator for another 2 hours at 37 °C. Then, the MTT solution was withdrawn and 100 μL of isopropanol was added to each well. Without waiting, the absorbance was read on a microplate reader with a 570 nm filter (reference wavelength 650 nm).

#### 2.4.4 Apoptosis/necrosis (double staining) assay

Test samples were prepared exactly as described in Section 2.4.3. Cells were seeded into 48-well culture plates at a density of 15 × 10^3^ cells *per* well and incubated for 24 h at 37 °C in a humidified atmosphere containing 5% CO_2_ (>90% humidity). Following initial attachment, the culture medium was aspirated, and the 50% diluted extract was added to the wells. Untreated cells served as the negative control, and all cultures were incubated for an additional 48 h. At the end of the treatment period, the medium was removed, and 70 µL of the double-staining solution was added to each well. The plates were then incubated for 15 min in the dark. Finally, apoptotic and necrotic cells were evaluated via FITC fluorescence (excitation/emission wavelength range of 480–520 nm) using a DAPI filter on an inverted fluorescence microscope (Leica DM6000B, Germany).

Stock solutions were prepared using Ribonuclease (RNase) (10 mg/1 mL PBS), Hoechst 33,342 (200 µg/1 mL PBS), and propidium iodide (PI) (100 µg/1 mL PBS). Subsequently, the dual staining solution was prepared by mixing 100 µL of RNase stock, 500 µL of Hoechst stock, and 100 µL of PI stock into 10 mL of PBS. During the procedure, 79 µL of this solution was added to each well, followed by a 15-min incubation. After a washing step, the samples were examined under a fluorescence microscope for apoptosis and necrosis.

#### 2.4.5 Scratch assay

Test samples were prepared exactly as described in Section 2.4.3. This test method is a method used *in vitro* to detect scratch closure. Scratch closure and spreading abilities of cells were assessed using a scratch wound assay, which measures the expansion of a cell population on surfaces. Cells were planted in a 24-well cell culture dish at 2 × 10^5^ cells/mL and incubated at 37 °C for 24 h. After the cells became confluent and covered the plate, a linear wound sample was created on the plate in one go with a sterile 100 µL plastic pipette tip. The plates were washed with PBS to remove the shielded cells and medium residue. 100 μL of each formulation at a concentration of 0.25 mg/mL and only the medium was applied as the control group. Then, it was incubated at 37 °C containing 5% CO_2_. While the created wound scratch was incubated for 24 h, the wound areas were photographed at 0, 12, and 24 h.

*In vitro,* wound healing and scratch closure were calculated by measuring the areas at the time the images were taken using ImageJ, an open-source image processing program. The percentage of wound closure was calculated according to the following Equation 1:

$$\%\text{ Closure}=\left(\text{initial scratch area }–\text{ scratch area measured at time }t\right)\;/\text{ initial scratch area}\times 100$$ (1)

#### 2.4.6 Antibiogram assay

Test microorganisms were initially incubated in nutrient broth at 37 °C for 2 h. Once turbidity developed, the suspensions were adjusted to a 0.5 McFarland standard (corresponding to approximately 10^8^ colony-forming units *per* mL). Erythromycin (15 μg) was used as the reference antibiotic for *Staphylococcus aureus* (ATCC 25923) and *Escherichia coli* (DH10B). Using sterile swabs, the standardized bacterial suspensions were evenly inoculated onto the surface of Mueller-Hinton agar plates. Subsequently, sterile antibiotic disks were applied, and blank paper disks impregnated with 30 µL of the 1 mg/mL formulation solutions were placed onto the agar. Disks were spaced at least 22 mm apart with a 14 mm clearance from the Petri dish edge to prevent overlapping of inhibition zones. Finally, the plates were incubated at 37 °C for 24 h, after which the resulting inhibition zone diameters were measured.

### 2.5 Statistical analysis

All analyses were performed in GraphPad Prism 9 (GraphPad Software, San Diego, CA, United States of America). For each endpoint the experimental unit is defined as follows: for fiber diameter, the independent fabrication batch; for contact angle and tensile properties, the individual specimen, with specimens drawn from 3 independent batches; for the MTT, apoptosis/necrosis and scratch assays, the well, with wells drawn from 6 independent experiments performed on different days with separate cell passages; for the antibiogram, the plate, with 6 plates from 6 independent experiments. Repeated measurements within a single specimen, image or well were treated as technical replicates and averaged before analysis; they were never entered as independent observations.

Where the assumptions were met, contact angle, fiber diameter and tensile data were compared by one-way ANOVA with Tukey’s HSD *post hoc* test. MTT data were analysed by two-way ANOVA with formulation (7 levels: F1-F6 plus control) and concentration (5 levels) as fixed factors, including the formulation × concentration interaction term; apoptosis/necrosis data by two-way ANOVA with formulation (6 levels) and concentration (2 levels); scratch data by two-way ANOVA with formulation (7 levels) and time (2 levels); and antibiogram data by two-way ANOVA with formulation and organism as factors. Interaction terms were tested in every two-way model and are reported. *Post-hoc* comparisons used Tukey’s HSD, and adjusted p-values are given. Significance was set at p < 0.05. Complete *post hoc* output is provided in the Supplementary Material.

## 3 Results and discussion

### 3.1 Characterizations of electrospun nanofibers

All six solutions could be electrospun continuously without needle clogging or jet interruption. The resulting fibers differed markedly in morphology: the PCL-only nanofiber (F1) contained numerous beads along the fibers, whereas bead density decreased progressively with the addition of HA and of the extract (Section 3.1.3). The resulting nanofibrous scaffolds were subsequently characterized to determine their surface wettability and mechanical performance.

#### 3.1.1 SEM and fiber morphology

SEM was used to investigate the fiber diameters and surface morphology of the nanofibers. Figure 1 displays the surface morphology of the resultant nanofibers. The nanofiber surfaces with bead-like structures were visible in SEM images. As seen in Table 3, the diameter of the nanofibers produced with the polymer solution containing only PCL is smaller than in other formulations. As a result of the study, it was determined that the nanofibers obtained were in the range of 300–700 nm (Figure 2). The diameters of the produced PCL nanofibers are consistent with the research of Sivakumar et al. (Sivakumar et al., 2024). Additionally, nanofiber diameters increased with the addition of HA and *C. coggygria* extract to the formulation. When the SEM images were examined, a lot of bead formation was observed in the F1 formulation, but with the addition of HA and *C. coggygria* to the formulation, the bead formation disappeared.

**FIGURE 1:**
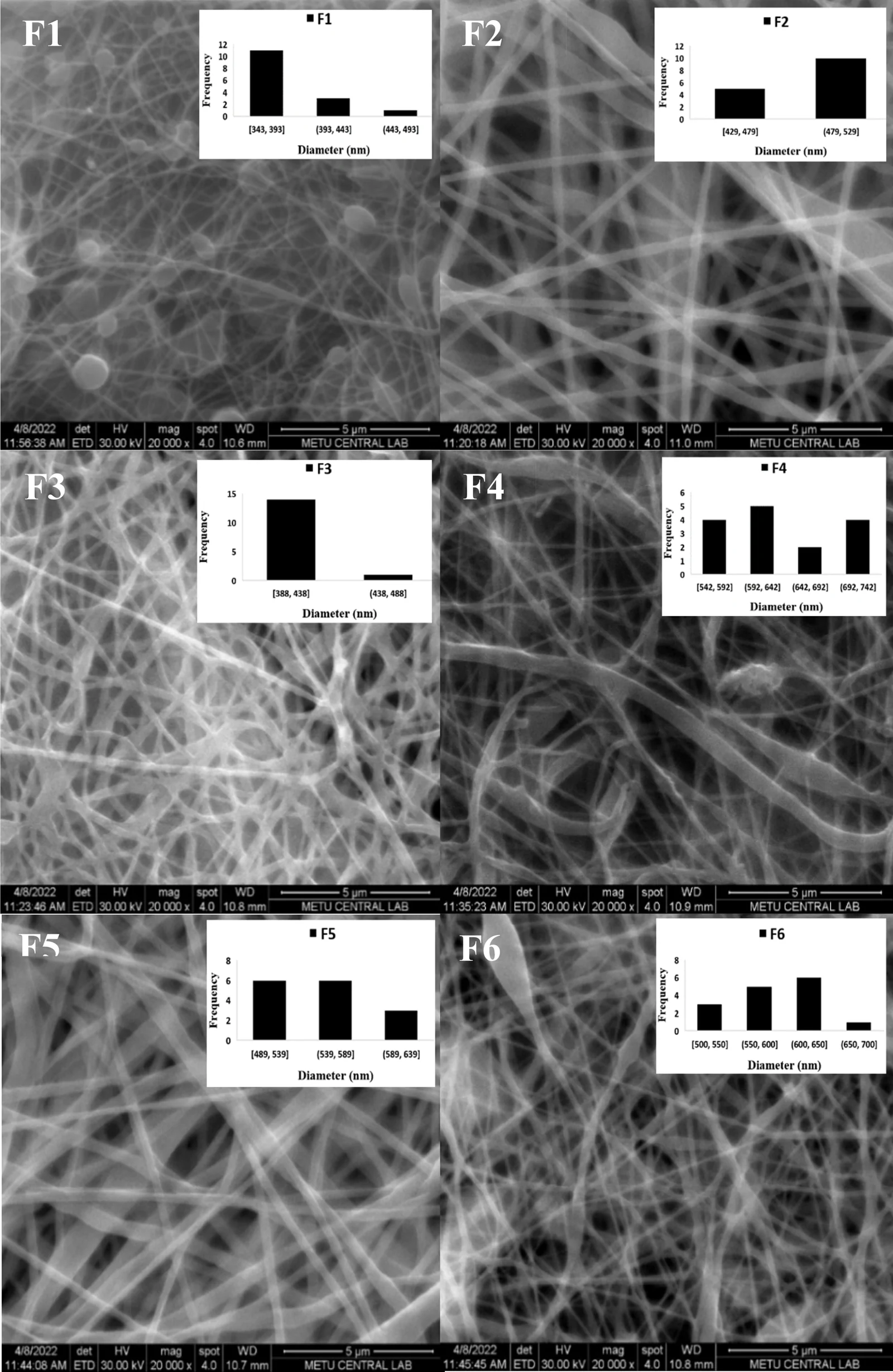
SEM images of formulations (F1: produced only with PCL, F2: produced with PCL and HA, F3 and F4: produced with PCL and *C. coggygria* extract at different concentrations, F5 and F6: produced with PCL, HA, and *C. coggygria* extract at different concentrations. Each SEM image is presented with its corresponding diameter distribution graph. (magnification ×20000 for each formulation).

**TABLE 3:** Mean diameters of the nanofibers (mean ± SD).

Formulations	Mean diameters (nm)
F1	390 ± 33
F2	481 ± 27
F3	420 ± 22
F4	635 ± 68
F5	548 ± 41
F6	593 ± 48

**FIGURE 2:**
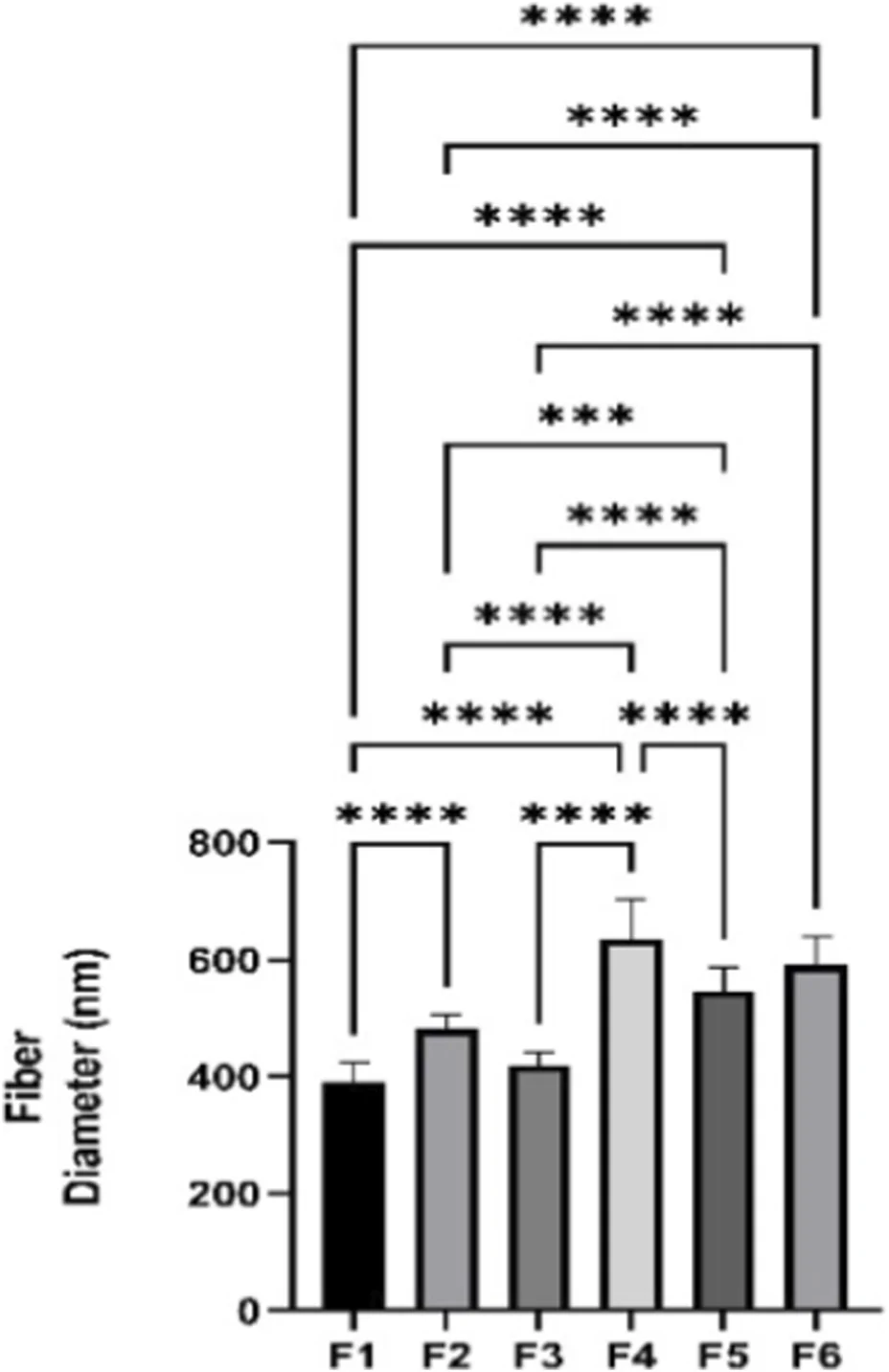
Graph of statistical analysis of fiber diameter obtained from SEM images (****: *p* < 0.0001, ***: *p* < 0.001). (Results of the one-way ANOVA comparing the formulations to one another).

Analysis of nanofiber diameter variations indicated that incorporating HA significantly increased fiber thickness in both the blank formulation and the 1% extract-loaded group (*p* < 0.05). Conversely, adding the extract alone to the blank formulation did not produce a statistically significant change in diameter. However, the combined presence of HA and the extract led to a pronounced increase in fiber diameter compared to the baseline F1 formulation (*p* < 0.05).

#### 3.1.2 Contact angle study

The results of the contact angle study of each formulation are given in Table 4. Images of the contact angle measurements are presented in Supplementary Figure S1. PCL is a highly hydrophobic polymer, thus, formulation F1 containing only PCL displayed the highest contact angle. As seen in Figure 3, the contact angle decreased, and so hydrophilicity increased with the addition of HA and *C. coggygria* extract to the formulation. The addition of *C*. *coggygria* extract in the formulation decreased the wettability dramatically. F6 formulation, which contains 3% *C. coggygria* and 0.5% HA, wetted rapidly, absorbed the complete water, and no drop was observed. The contact angle obtained in the F1 formulation is in accordance with the inherently hydrophobic nature of PCL. Due to this hydrophobic structure, PCL nanofibers do not become wet, and water droplets remain on the surface with minimal contact with the polymer (Khalid et al., 2025). A directly comparable trend was reported by Adamu et al. (Adamu et al., 2024), who incorporated *Croton macrostachyus* Hochst. ex Delile (CM) leaf extract into PCL nanofibers and observed a progressive reduction in contact angle with increasing extract concentration, achieving full hydrophilicity (contact angle ≈0°) at 7% extract loading. Similarly, demonstrated in PCL–cellulose acetate (PCL-CA)/CM nanofibers that incorporation of CM extract improved the swelling capacity and surface wettability of the mats, which directly correlated with enhanced wound exudate absorption *in vitro*. However, in the F2 formulation, which contains HA, the hydrophilic character of HA imparted wettability to the nanofiber, resulting in a decrease in the measured contact angle (Huang et al., 2025). Wettability was governed by the extract content. Increasing the extract from 1% to 3% reduced the contact angle from 51.5° ± 8.5° (F3) to 19.6° ± 5.5° (F4), whereas adding 0.5% HA to a PCL matrix produced only a marginal change (F1, 122° ± 3° vs. F2, 118° ± 1°) that was not statistically significant. Consistently, F5 (0.5% HA + 1% extract, 65.3° ± 3.3°) did not show a lower contact angle than F3 (1% extract alone). We attribute this to the fact that HA at 0.5% (w/v) is distributed within the fiber bulk rather than enriched at the fiber surface, and that the HA-containing solutions produced thicker fibers (Table 3), which reduces the specific surface area available for capillary uptake. Within the range studied, surface wettability was determined by the extract content. HA at 0.5% (w/v) did not produce a statistically significant change in contact angle either in the absence (F1 vs. F2) or in the presence (F3 vs. F5) of the extract. Accordingly, the notably lower contact angle observed in the F6 formulation containing HA and 3% extract was anticipated, reflecting the combined and cooperative influence of these hydrophilic constituents at elevated concentrations.

**TABLE 4:** Contact angle values of the formulations (n = 3) (mean ± SD).

Formulations	Contact angle (°)
F1	122 ± 3
F2	118 ± 1
F3	51.5 ± 8.5
F4	19.6 ± 5.5
F5	65.3 ± 3.3
F6	droplet fully absorbed in 3 s

**FIGURE 3:**
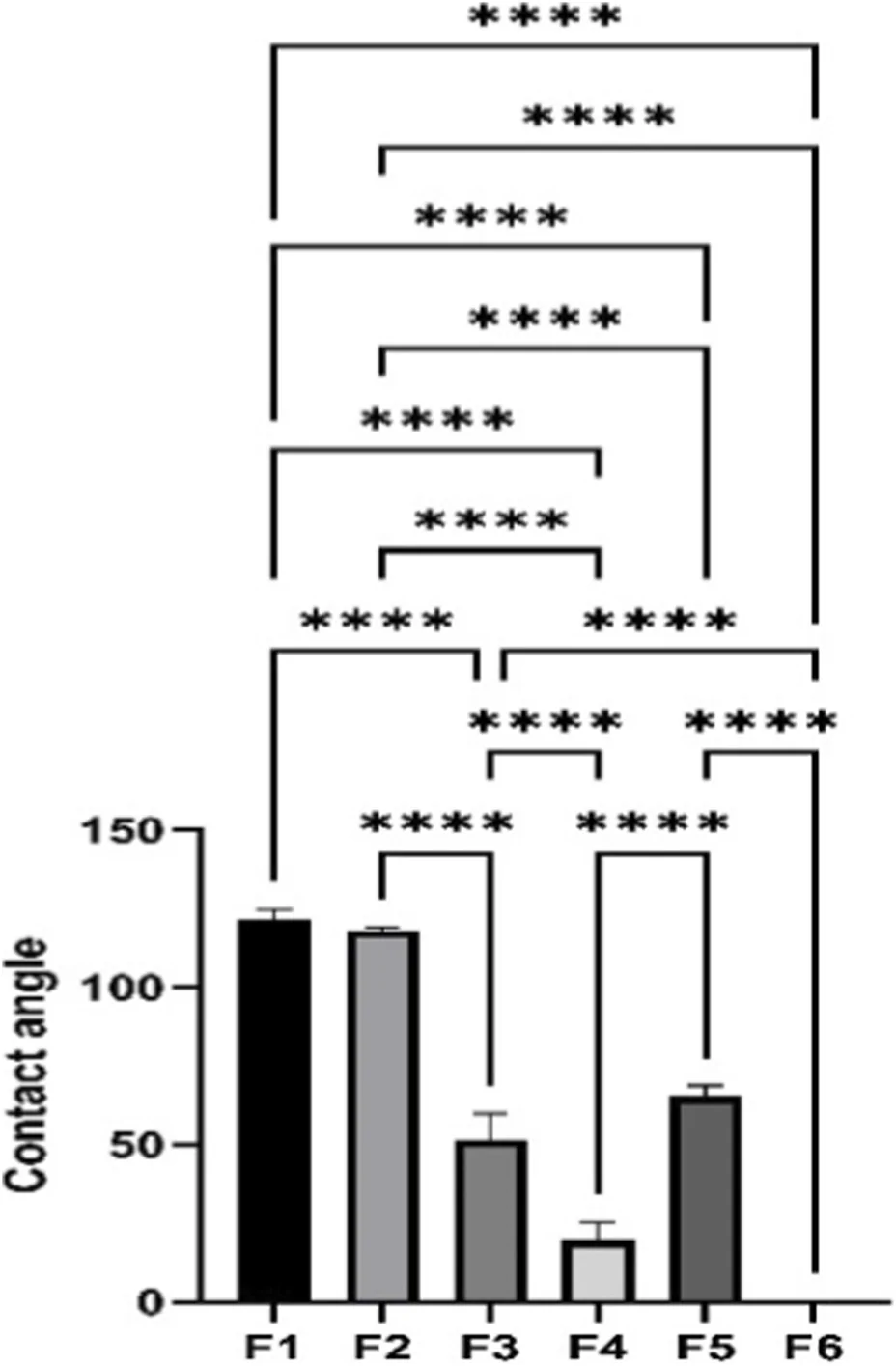
Graph of statistical analysis of contact angle results (****: *p* < 0.0001) (Results of the one-way ANOVA comparing the formulations to one another).

Statistical analysis indicated that both the incorporation and concentration of the extract significantly influenced the surface contact angle (*p* < 0.05). In contrast, the addition of HA did not yield a statistically significant alteration in surface wettability.

#### 3.1.3 Mechanical properties

Elongation at break and tensile strength values of the formulations are given in Table 5. Representative tensile strength curves are included in the supplementary data. In F1 formulation tensile strength and elongation at break values were lower than obtained results in similar studies (Shafiei et al., 2016; Fadaie et al., 2018). This could be caused by the beaded nanofibers in formulations. Our results were close to Khunová et al.’s study, as it was seen beaded structure changed the texture results (Khunova et al., 2022). A comparison between the F1 and F3 formulations indicates that incorporating the extract into the PCL matrix enhanced both tensile strength and elongation at break (Figure 4). Conversely, among the ternary formulations containing PCL, HA, and *C. coggygria* extract, increasing the extract concentration led to a progressive reduction in these mechanical properties. F6 was the composition with the highest extract load (3%) in the three-solvent system, and its fibers exhibited visible local variation in areal density; specimens sampled from different regions of the same fibers therefore differed substantially in effective cross-section. The mean values for F6 should accordingly be treated as indicative only, and F6 is not proposed as a lead composition on mechanical grounds. F5, which combined the highest tensile strength (2.40 ± 0.15 MPa) with the highest elongation at break (178% ± 18%) and low variability, is the composition carried forward. By contrast, Kumelachew et al. (Kumelachew et al., 2025) reported substantially higher tensile strengths (7.8 ± 0.52 MPa vs. 4 ± 0.24 MPa for core-only PCL fibers) in polyurethane (TPU)/PCL core-shell nanofibers reinforced with calcium carbonate nanoparticles—a mechanically optimized system designed for colorectal stent cover applications where load-bearing capacity is paramount. Because ideal wound-dressing materials require a balance of sufficient mechanical strength for durability and adequate elasticity for ease of application, the F5 and F6 formulations demonstrated the most suitable overall mechanical profile for wound-healing applications. As reported in the study by Meng et al., PCL concentrations at or below 10% have been shown to provide superior mechanical properties. In accordance with these findings, a 20% PCL concentration was selected for microfiber production, while concentrations of 10%, 6%, and 2% were used for the nanofibers, yielding integrated composite structures. Mechanical evaluations demonstrated that the 2% PCL formulation achieved the most favorable balance of tensile strength and elongation at break. These promising outcomes provide a solid rationale for investigating a broader range of PCL concentrations in future studies (Meng et al., 2024). The mechanical response of F6 was markedly heterogeneous (RSD >55% for both parameters).

**TABLE 5:** Tensile strength and elongation at break values of the formulations (n = 3) (mean ± SD).

Formulations	Tensile strength (MPa)	Elongation at break (%)
F1	0.388 ± 0.078	38.3 ± 19.3
F2	0.945 ± 0.081	29.0 ± 9.8
F3	2.16 ± 0.23	53.8 ± 5.2
F4	1.01 ± 0.49	61.5 ± 12.7
F5	2.40 ± 0.15	178 ± 18
F6	1.70 ± 1.03	99.9 ± 56.6

**FIGURE 4:**
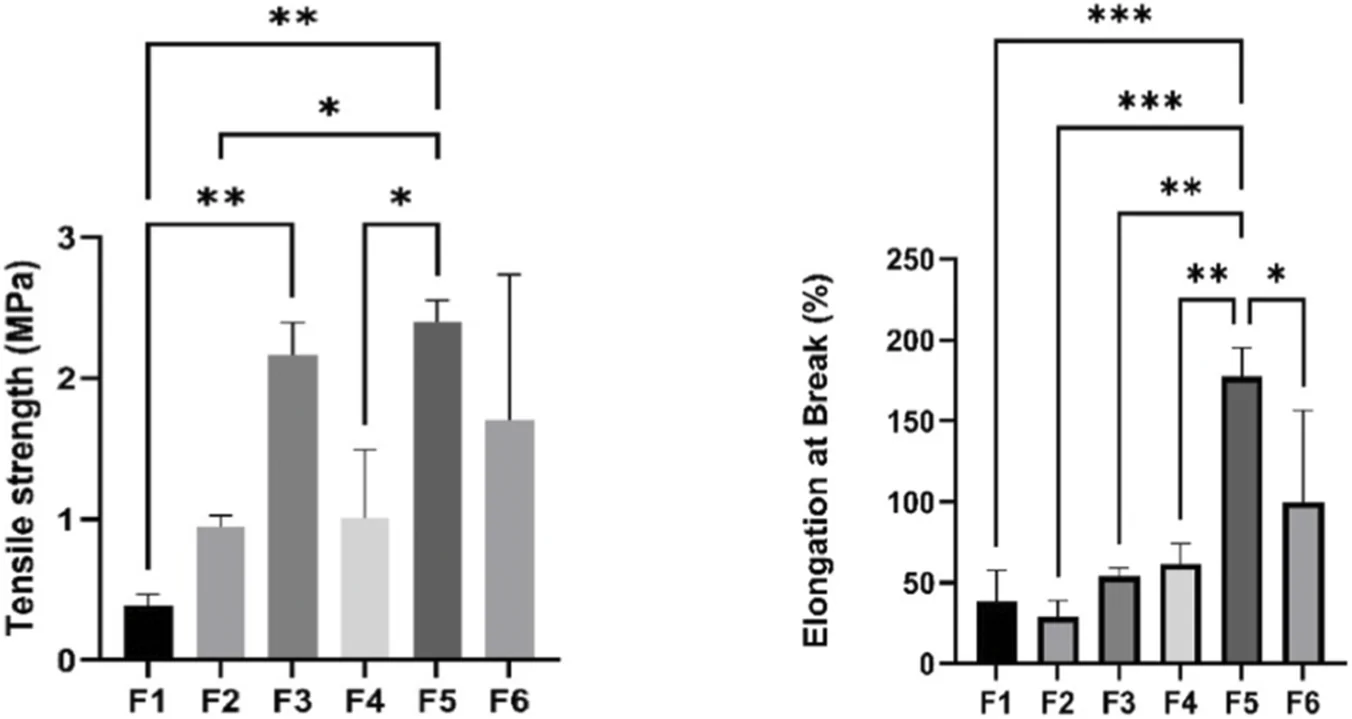
Graphs of statistical analysis of mechanical properties (Tensile strength and elongation at break) results (***: *p* < 0.001, **: *p* < 0.01, *: *p* < 0.05). (Results of the one-way ANOVA comparing the formulations to one another).

Statistical evaluation revealed that incorporating *C. coggygria* extract significantly affected tensile strength (*p* < 0.05), whereas the addition of HA alone produced no significant effect on this parameter. Conversely, alterations in elongation at break were statistically significant when HA was added to formulations containing the extract but remained unaffected in the blank formulation. Notably, the F5 formulation comprising 1% extract combined with HA exhibited a statistically significant difference across all parameters compared to every other tested formulation. (*p* < 0.05).

#### 3.1.4 DSC, FT-IR and Confocal Raman spectroscopy analyses

The DSC thermogram, FT-IR spectra and Raman spectra were presented in respectively Figures 5–7.

**FIGURE 5:**
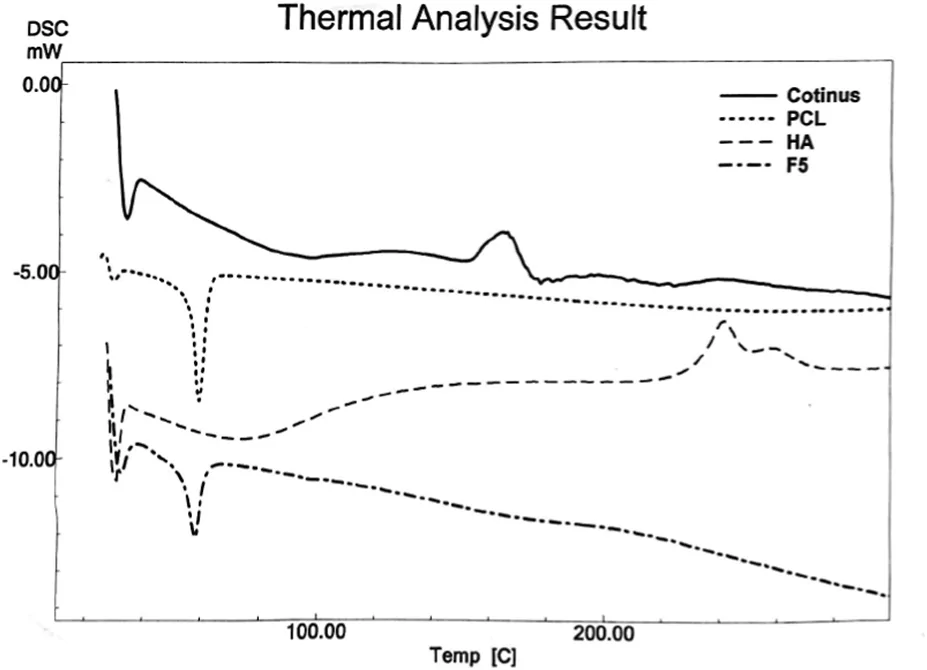
DSC thermogram of *C*. *coggygria* extract, PCL, HA, and F5 formulation. DSC analysis revealed the distinctive endothermic peak of PCL at 60 °C and the distinctive exothermic peak of HA at 240 °C (Batool et al., 2025; Gedik and Erdem, 2025). No DSC analysis showing specific data for *C*. *coggygria* extract was found in the literature. The DSC results are consistent with the FT-IR analysis. In this analysis, as in FT-IR, all components exhibited specific peaks, but since the highest concentration in the F5 formulation belonged to PCL, only the peak belonging to PCL was detected in the F5 formulation.

**FIGURE 6:**
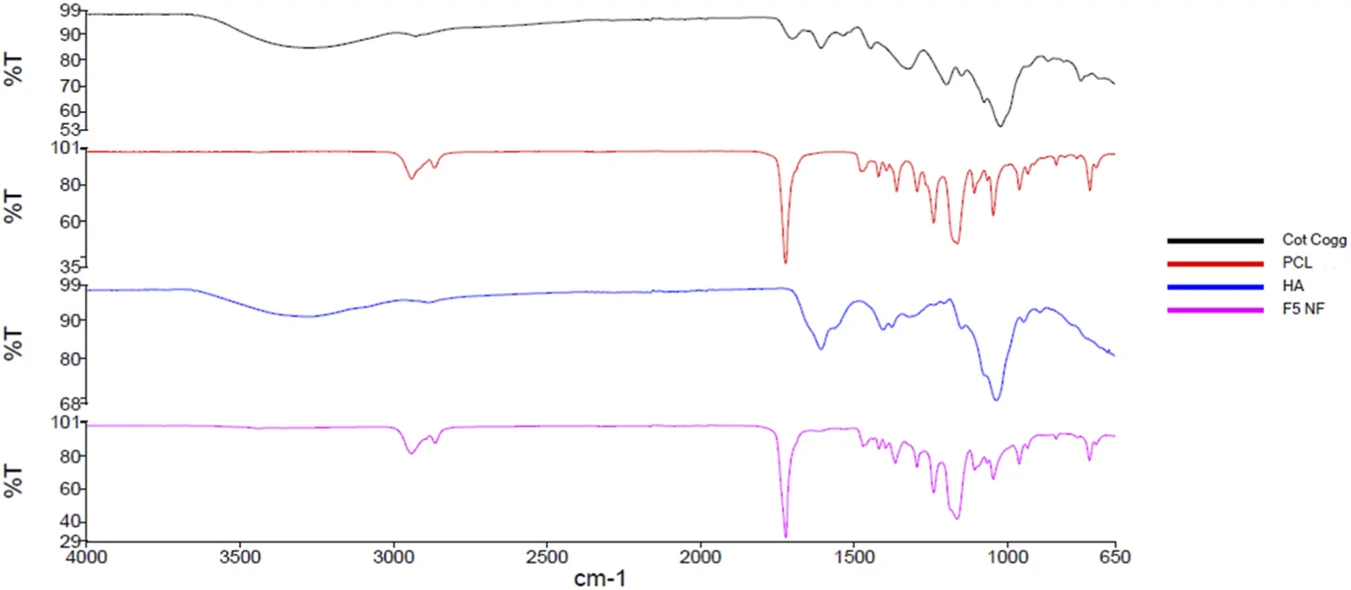
FT-IR spectra of *C*. *coggygria* extract, PCL, HA, and F5 formulation. The infrared bands at 2,949 cm^-1^, 1732 cm^-1^, 1,296 cm^-1^ and 1,172 cm^-1^, which are the characteristic peaks indicating asymmetric CH_2_ stretching, C=O carbonyl stretching, C-O and C-C stretching in the crystalline phases and symmetric C-O-C stretching in the structure of PCL, were observed in both PCL and F5 formulation (Eskitoros-et al., 2019). HA-specific peaks were found at 1,628 cm^-1^, 1,386 cm^-1^, and 1,047 cm^-1^ (Tsanaktsidou et al., 2019). FT-IR analysis revealed characteristic peaks of *Cotinus coggygria* extract at 3,200 cm^-1^, 2,900 cm^-1^, and 1,200 cm^-1^ (Rahman et al., 2022). All of the compounds segregate their own distinct bond peaks in FT-IR spectral analysis; however, because of the high percentage of PCL in the F5 formulation, peaks corresponding to HA or *C*. *coggygria* extract were not detectable.

**FIGURE 7:**
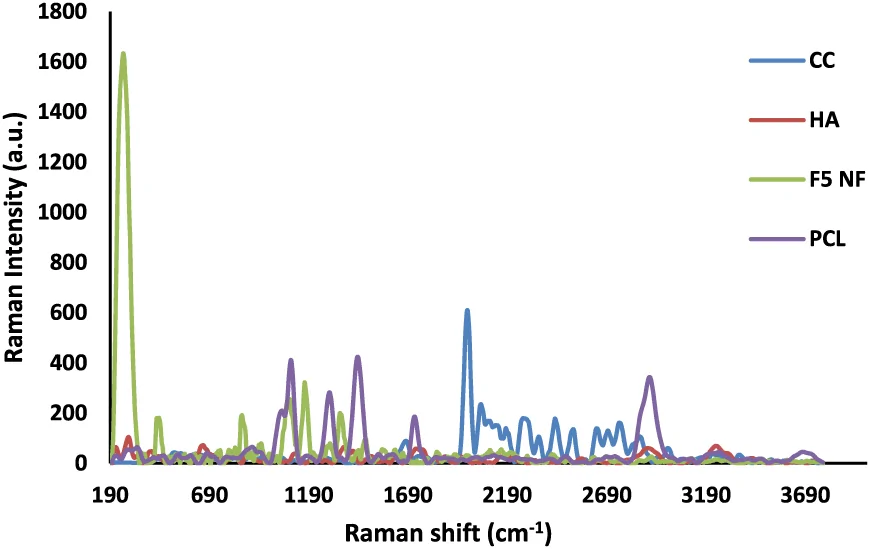
Confocal Raman spectra of *C*. *coggygria* extract, PCL, HA, and F5 formulation. In the Raman spectrum, characteristic peaks of 2,916.5 cm^-1^, 1727.5 cm^-1^ and 1,443 cm^-1^ belonging to PCL and 1,121 cm^-1^, 2,911 cm^-1^ and 3,418 cm^-1^ belonging to HA were detected (Smith et al., 2017; La Verde et al., 2022; Ito et al., 2010). It was concluded that there was an overlap of particular PCL peaks in the Raman spectrum as in DSC and FT-IR since PCL was the compound present in the highest concentration in the F5 formulation.

### 3.2 Cell culture studies

#### 3.2.1 Cell viability assay

The results of the MTT assay assessing mitochondrial activity are summarized in Table 6. L929 mouse fibroblasts were used because they are the reference cell line recommended for the cytotoxicity testing of medical devices under ISO 10993-5. They are an immortalized subcutaneous connective-tissue fibroblast line and are not intended to model the cellular composition of human dermis. The MTT assay reports mitochondrial dehydrogenase activity and is used here strictly as an indicator of cytotoxicity. The data indicate that the toxic effects of the nanofibers are concentration-dependent, with formulations F1, F4, and F6 exhibiting notable cytotoxicity at higher concentrations (0.5 and 1 mg/mL). In contrast, formulations F3 and F5 remained non-toxic across all tested doses Figure 8. Overall, these findings suggest that most tested formulations are safe up to a concentration of 0.5 mg/mL, with the exception of F4. As seen in the research of Fadaie et al., PCL is a non-toxic polymer (Fadaie et al., 2018). However, the F1 formulation exhibited cytotoxicity at a dose of 1 mg/mL. Conversely, incorporating *C. coggygria* extract into the formulation mitigated cytotoxicity in L929 cells, a finding that aligns with previous observations reported by Bozkaya et al. (Bozkaya et al., 2023). Pure PCL nanofibers exhibited measurable cytotoxicity, whereas the incorporation of HA mitigated these adverse effects due to its inherent cell-proliferative properties. The effect of the extract on L929 viability was biphasic. At 1% (F3), viability at 1 mg/mL was higher than that of the PCL-only fiber (71.8% ± 2.5% vs. 44.9% ± 2.1%); at 3% (F4) it was lower (33.1% ± 1.1%). The same pattern was observed at 48 h in the apoptosis/necrosis assay, where F4 gave the highest apoptotic (21% ± 1%) and necrotic (39% ± 3%) indices of any fiber, and in the scratch assay, where F4 gave the lowest closure (24% ± 3% at 24 h). Polyphenol-rich extracts are widely reported to show this pattern, being cytoprotective at low concentrations and cytotoxic at high ones. We therefore do not claim that the extract reduces cytotoxicity; we report that 1% extract was well tolerated whereas 3% was cytotoxic, and that this dose dependence is the principal reason for selecting F5 rather than F6 as the lead composition. Furthermore, the inclusion of *C. coggygria* extract enhanced cell viability, a protective effect facilitated by its rich profile of flavonoids and tannic compounds. Consequently, the optimized F5 and F6 preparations displayed minimal cytotoxicity, although the viability observed with F6 remained dose-dependent (Bozkaya et al., 2023; Sivakumar et al., 2024; Samavati et al., 2023). The MTT assay results revealed statistically significant differences in cell viability across all formulations and concentrations compared to both positive and negative controls (*p* < 0.05), with a few notable exceptions where no significant variations were detected. Specifically, no significant differences were found between the positive control and the 1 mg/mL F4 formulation, or between F1 and F6 at 0.5 mg/mL. Similarly, comparable viability levels were observed between the negative control and F5, as well as between F2 and F6 at 0.25 mg/mL, and between F3 and F6 at 0.125 mg/mL. At the lowest tested concentration of 0.062 mg/mL, parity was likewise noted between the negative control and F4, and between F3 and F4.

**TABLE 6:** % viability according to MTT test results on L929 normal fibroblast cell line. (Each formulation was extracted in the medium and cells were treated with different concentrations of each formulation solution).

Samples	% viability at tested concentrations*				
​	1 mg/mL	0.5 mg/mL	0.250 mg/mL	0.125 mg/mL	0.062 mg/mL
F1	44.9 ± 2.1	60.6 ± 3.5	68.0 ± 1.2	72.1 ± 2.8	80.1 ± 1.3
F2	63.7 ± 3.2	69.9 ± 2.2	74.8 ± 1.4	81.9 ± 3.1	88.3 ± 1.2
F3	71.8 ± 2.5	77.7 ± 2.6	88.9 ± 1.1	92.2 ± 3.2	95.5 ± 1.6
F4	33.1 ± 1.1	45.5 ± 3.8	64 ± 2	76.6 ± 3.1	96.3 ± 1.7
F5	80.4 ± 3.8	92.4 ± 1.1	97.8 ± 1.3	105 ± 3	109 ± 1
F6	58 ± 2	63.0 ± 3.2	73.7 ± 1.6	90.9 ± 3.6	110 ± 1

* Viability was obtained as 100% in negative control (medium only), and 35% ± 2.4% in positive control (20% DMSO).

**FIGURE 8:**
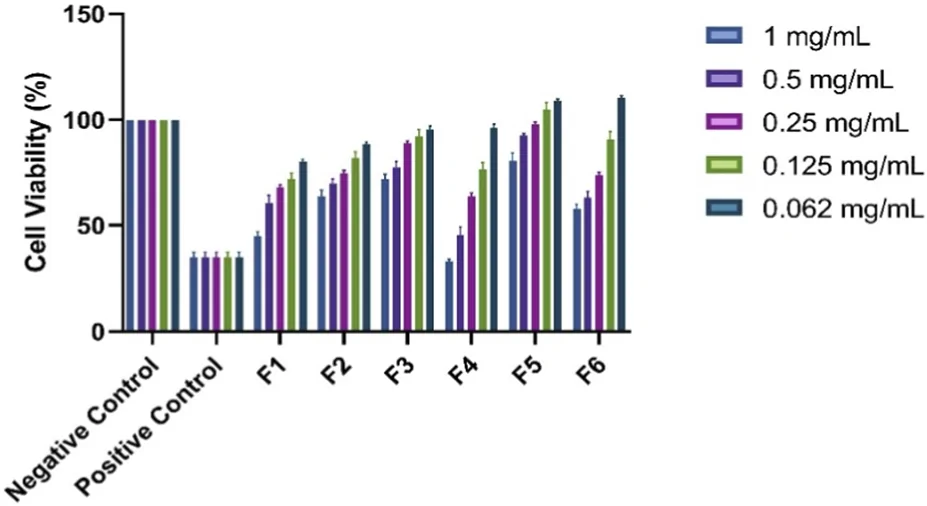
Statistical analysis graph of % Cell Viability from MTT assay on L929 normal fibroblast cell line. Cells were treated with different concentrations (1 mg/mL, 0.5 mg/mL, 0.25 mg/mL, 0.125 mg/mL, 0.062 mg/mL) of each formulation solution. (Results of the two-way ANOVA comparing the formulations with negative control and positive control groups).

The absence of significant differences between certain formulations and the negative control indicates a lack of appreciable cytotoxicity. Nevertheless, the sporadic distribution of statistical significance across varying doses and formulations coupled with the absence of a clear, dose-dependent trend suggests that some of the observed variations may stem from inherent experimental variability, warranting further investigation to confirm these findings.

#### 3.2.2 Apoptosis/necrosis (double staining) assay

Table 7 summarizes the apoptosis and necrosis rates determined via double staining. These cell death profiles closely mirrored the cytotoxicity trends observed earlier, with the F4 formulation eliciting the highest rates of both apoptosis and necrosis, as further illustrated in Figure 9. As seen in the images, cell death was observed to occur mostly in the necrotic way Figure 10. The fact that necrosis was higher than apoptosis in all formulation samples shows that cell deaths are not planned but caused by the applied formulation. When examining the percentages of apoptosis and necrosis, similar to the MTT study, the addition of *C. coggygria* extract initially reduced apoptosis and necrosis. The inclusion of HA in comparable extract-containing formulations (F5, F6) resulted in a notable reduction in both apoptosis and necrosis, consistent with HA’s well-documented anti-inflammatory and cell-survival-promoting activities through receptor-mediated signalling (Astaneh and Fereydouni, 2023; Huerta-Ángeles and Mixcoha, 2025). The addition of *C. coggygria* extract at low to moderate concentrations (F3, F5) reduced apoptotic and necrotic indices relative to pure PCL formulations (F1), reflecting the extract’s cytoprotective properties—an effect attributed to the scavenging of reactive oxygen species by polyphenolic and flavonoid constituents, which are particularly abundant in *C. coggygria* leaf extract. This observation is mechanistically consistent with the antioxidant and anti-inflammatory pharmacology of *C. coggygria* reported in the literature and with findings from comparable plant extract-loaded nanofiber systems, including those incorporating CM and R. prinoides extracts (Adamu et al., 2024), where the polyphenolic fractions of the plant extracts similarly contributed to reduced cellular oxidative stress. However, increasing its concentration in the formulation (F6) led to higher levels of apoptosis and necrosis compared to the F5 formulation. In the % apoptosis analysis at a concentration of 1 mg/mL, no statistically significant differences were observed between the following formulation pairs: F1 and F4, F2 and F6, and F3 and F5. Similarly, at a concentration of 0.5 mg/mL, no significant differences were found between F1 and F6, F2 and F3, F2 and F5, and F3 and F5. Beyond these specific comparisons, no other statistically significant differences were observed among the remaining formulations for % apoptosis.

**TABLE 7:** % Apoptotic and necrotic index values (n = 6) (mean ± SD).

Samples	Apoptosis (%)		Necrosis (%)	
​	1 mg/mL	0.5 mg/mL	1 mg/mL	0.5 mg/mL
F1	20 ± 0	10 ± 1	21 ± 2	15 ± 0
F2	12 ± 0	6 ± 1	15 ± 0	2 ± 0
F3	7 ± 0	6 ± 1	5 ± 0	3 ± 0
F4	21 ± 1	12 ± 1	39 ± 3	28 ± 2
F5	6 ± 1	5 ± 1	10 ± 1	8 ± 1
F6	11 ± 2	9 ± 1	12 ± 2	10 ± 1

**FIGURE 9:**
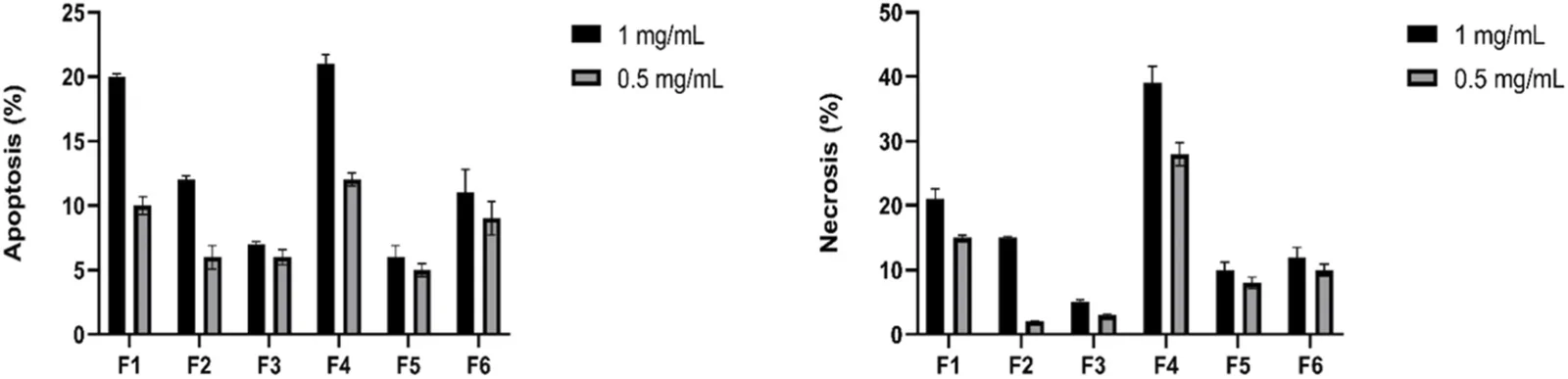
Statistical analysis graph of % apoptosis and % necrosis. Different concentrations (1 mg/mL, 0.5 mg/mL) of each formulation were applied to the well. (Results of the two-way ANOVA comparing the formulations to one another).

**FIGURE 10:**
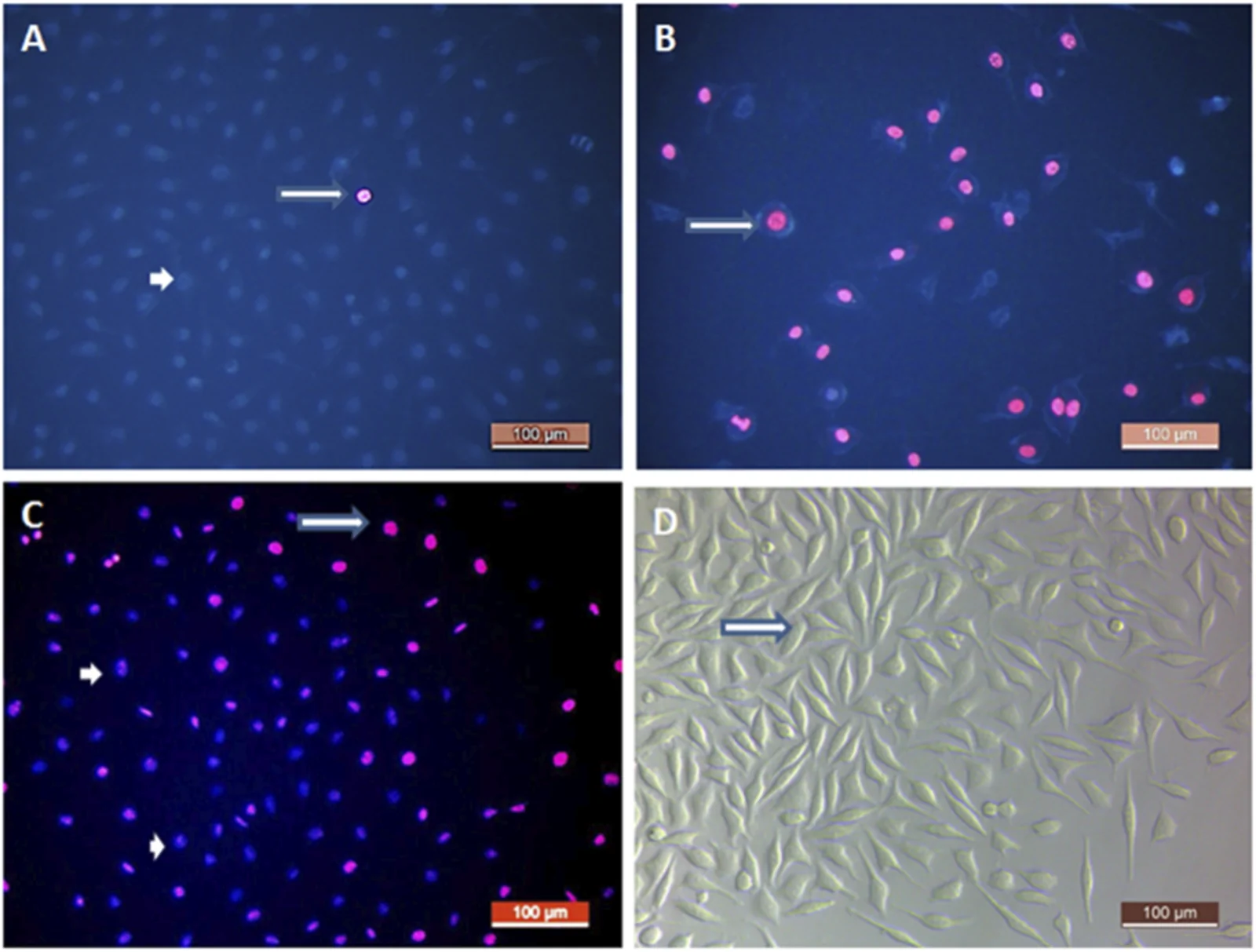
Fluorescence microscopy images of formulations applied on L929 (fibroblast) cells. **(A)** Photograph of L929 cells without formulation application obtained by the dual staining method (long arrow indicates necrotic, short arrow indicates normal healthy cells). **(B)** Photograph showing necrosis in cells treated with 1 mg/mL F4 formulation (the long arrow indicates necrotic red cells). **(C)** Photograph showing apoptosis and necrosis together in cells treated with 1 mg/mL F4 formulations (long arrow shows cells with necrotic red nuclei and the short arrow shows cells with apoptotic blue nuclei). **(D)** Light microscope photograph of F4 formulation immature fibroblast cells. Photos were taken with a Leica DM6000 fluorescence microscope. The scale of images is 100 µm.

These results suggest that incorporating *C. coggygria* extract contributes to apoptotic activity within the formulations. Furthermore, while adding HA did not yield a significant difference in the percentage of apoptosis between F3 and F5, its inclusion led to a notable reduction in apoptosis when comparing the F4 and F6 formulations. At the 1 mg/mL concentration, necrosis percentages showed no statistically significant difference between formulations F5 and F6, whereas all other formulation pairs exhibited significant variations. Similarly, at 0.5 mg/mL, necrosis levels remained comparable between F5 and F6 consistent with the higher-dose findings as well as between formulations F2 and F3.

#### 3.2.3 Scratch assay

Since the biological process of wound healing is intricate, there are numerous *in vitro* and *in vivo* assays available. The scratch assay is one of these that has shown to be a useful and affordable method for gaining an early understanding of how preparations can favorably affect the formation of new tissue (Fronza et al., 2009). This is a widely used assay that is low-cost, simple to use, and involves mechanically creating an artificial scratch on a confluent cell monolayer by scratching off a portion of the cell layer. In this assay, a linear scratch is created across a confluent monolayer of cultured cells. Scratch closure rate is then quantified by capturing periodic images to monitor the progressive decrease in the width of the cell-free gap over time, as cells at the borders proliferate and migrate inward until the wound is fully closed (Ascione et al., 2016; De Ieso and Pei, 2018).

*In vitro* wound healing test results are given in Table 8. In addition, images of cells to which F2 formulation was applied and only medium was applied are given in Figures 11, 12 as an example photograph. As in the table, it can be seen that the scratch area exhibited the highest closure in the F2 group (Figure 13). It can be seen that the least closure occurs in formulation number F4. Figure 12 shows the scratch closure over time. While a 10% closure was observed in the control group at the end of 24 h, a closure of over 40% was observed in formulation-applied cells, except for F4. HA addition to pure PCL nanofibers (F2) and F3 and F4 formulations increased the % wound closure. Moreover, an increase in *C. coggygria* extract concentration decreased the % wound closure. According to these results, we can say that the % wound closure results are compatible with the cytotoxicity study. In the wound closure assay, no statistically significant differences were observed among formulations F1 and F5, F3 and F4, F3 and F6, and F4 and F6 after 12 h of treatment. By 24 h, this parity persisted only between F1 and F5. Taken together, all tested formulations significantly enhanced wound closure compared to the control group at both time points, with the overall therapeutic effect becoming more pronounced at 24 h.

**TABLE 8:** *In vitro* wound closure percentages in formulation-treated fibroblast cells.

Formulations	% wound closure	
​	12 h	24 h
F1	24 ± 1	58 ± 2
F2	31 ± 2	71 ± 3
F3	12 ± 1	47 ± 2
F4	13 ± 2	24 ± 3
F5	22 ± 2	57 ± 3
F6	15 ± 1	39 ± 2
Control	4.8 ± 5.3	9.5 ± 3.2

**FIGURE 11:**
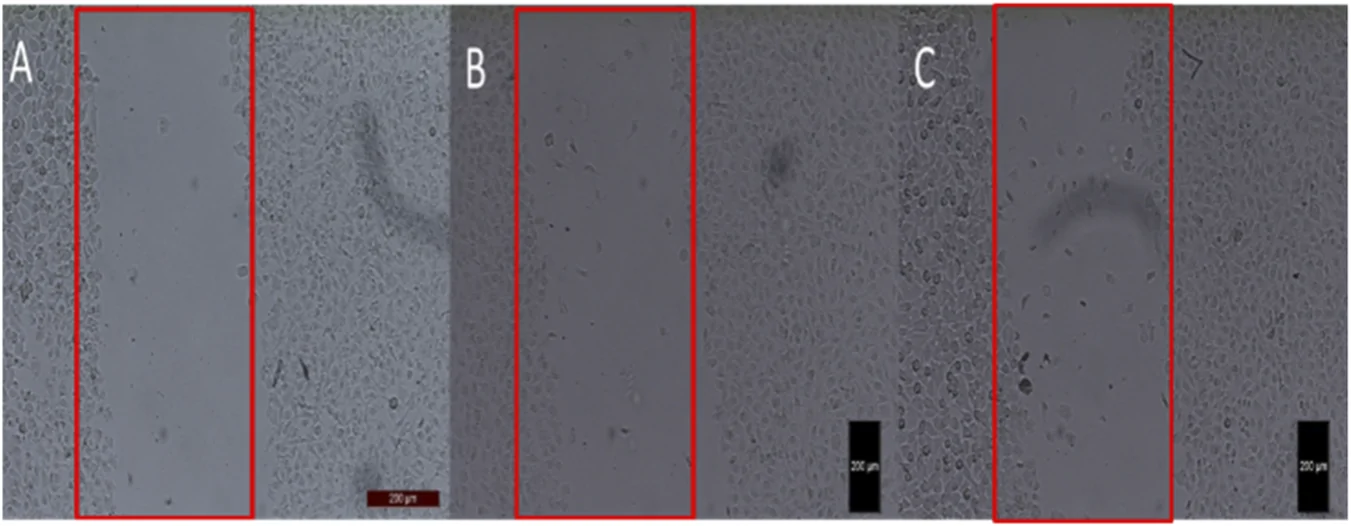
Microscope images of the scratch test of non-formulation-treated L929 (fibroblast) cells, marked areas show the wound areas. **(A)** 0^th^ hour, **(B)** 12th hour, **(C)** 24th hour).

**FIGURE 12:**
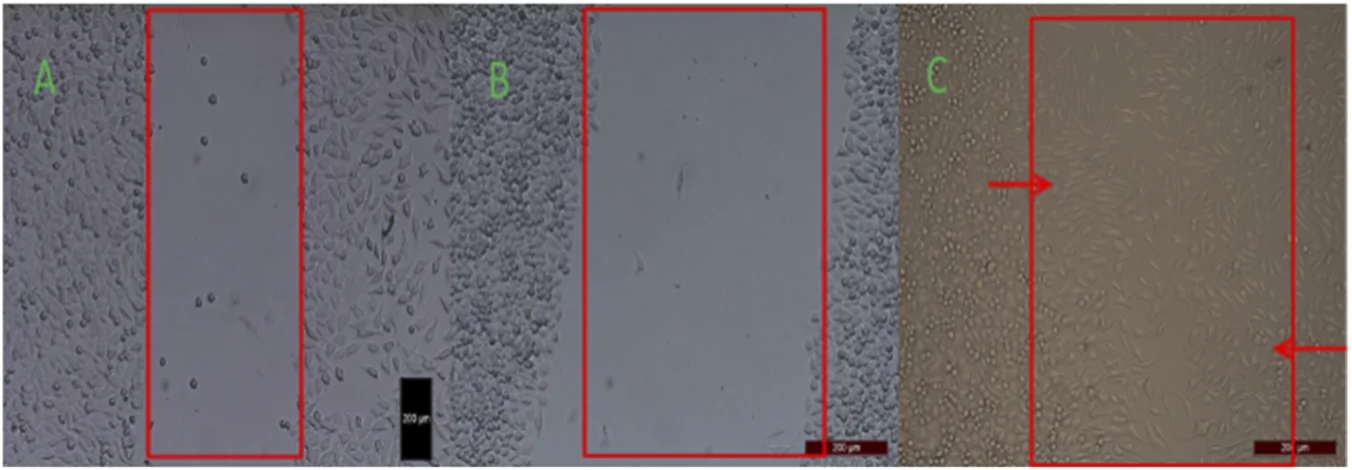
Microscope images of scratch test of L929 (Fibroblast) cells with F2 formulation (0.25 mg/mL applied), marked areas show the wound area. **(A)** 0^th^ hour, **(B)** 12th hours, **(C)** 24th hour, arrow mark indicates the area of closure in the wound area).

**FIGURE 13:**
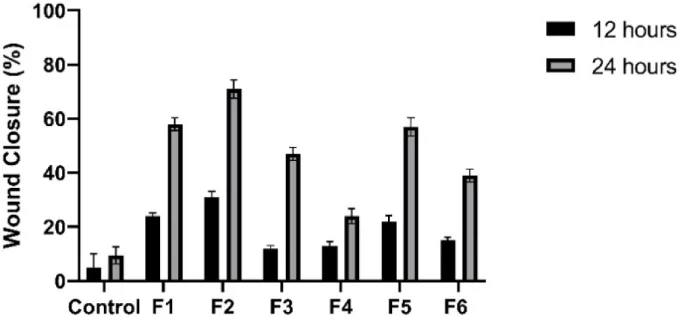
Statistical analysis graph of % wound closure obtained from scratch assay. Each formulation was applied to cells up to 12 (grey) and 24 (black) hours. (Results of the two-way ANOVA comparing the formulations with control group).

#### 3.2.4 Antibiogram assay

The antibacterial evaluation results obtained via the disc diffusion method are summarized in Table 9, with representative culture images presented in Figure 14. Because the inhibition zone diameter reflects the extent of bacterial growth inhibition, formulations were anticipated to yield zones comparable to or exceeding those of the reference antibiotic, erythromycin. The results demonstrate that at a concentration of 1 mg/mL, the formulations effectively inhibited *Staphylococcus aureus*, showing efficacy approaching that of the standard antibiotic, whereas their activity against *Escherichia coli* was less pronounced (Figure 15). Overall, formulations F1, F2, and F5 displayed notable antibacterial activity specifically against *S. aureus*. Among these three formulations, the F1 formulation exhibited a measurable effect despite lacking both HA and the plant extract, an outcome likely attributable to residual processing solvents retained during production. Although previous literature indicates that HA possesses inherent antibacterial activity against *E. coli* and *S. aureus*, the inclusion of HA in our study resulted in lower antibacterial activity compared to the pure PCL control formulation (Della Sala et al., 2022). Incorporating HA into the F3 and F4 formulations enhanced antibacterial activity against *S. aureus* without significantly altering efficacy against *E. coli*, an outcome attributed to the bioactive contribution of the *C. coggygria* extract. In our study, *C. coggygria* extract was prepared from leaves, and in the study of Matic et al., it was observed that the extract obtained from leaves had a higher antibacterial activity against *S. aureus* (Antal et al., 2021). These findings align well with existing literature. In the antibiogram assay against *S. aureus*, each formulation was evaluated relative to erythromycin. Statistical analysis revealed that only the F1 formulation exhibited no significant difference in antibacterial activity compared to erythromycin, whereas all other formulations exhibited significantly lower efficacy (*p* < 0.05). Interestingly, F1, which achieved performance comparable to the reference antibiotic, lacked both HA and *C. coggygria* extract. This unexpected activity is hypothesized to stem from residual processing solvents, which may have simultaneously contributed to the formation of bead-like morphological structures. Upon examination of the formulations, it was observed that the addition of HA to the extract-containing formulations significantly increased the inhibition zone diameter. Within the extract-containing series, the fibers also containing HA gave larger inhibition zones against *S. aureus* than their HA-free counterparts (F3 vs. F5; F4 vs. F6). Because the HA-containing and HA-free fibers were produced with different solvent systems, this difference cannot be assigned to HA alone and no interaction between HA and the extract is claimed. Evaluation of antibacterial activity against *E. coli* revealed that all formulations produced significantly smaller inhibition zones than erythromycin (*p* < 0.05), an outcome likely influenced by applied dosage differences. Taken together, these findings demonstrate that the developed nanofiber formulations possess greater antibacterial efficacy against *S. aureus* than against *E. coli*.

**TABLE 9:** Antibacterial effect of formulations and antibiotics against *Staphylococcus aureus* and *Escherichia coli*. All results are presented as the mean ± SD of six independent measurements.

Bacteria	*S. aureus* (zone diameters)		*E. coli* (zone diameters)	
Samples	A (erythromycin)	Sample	A (erythromycin)	Sample
F1	28 ± 1	27 ± 1	10 ± 1	8 ± 0
F2	24 ± 1	20 ± 0	11 ± 0	7 ± 0
F3	24 ± 1	15 ± 2	11 ± 1	6 ± 0
F4	26 ± 1	9 ± 1	10 ± 1	7 ± 0
F5	23 ± 1	21 ± 0	10 ± 1	6 ± 1
F6	17 ± 2	11 ± 1	11 ± 1	6 ± 0

**FIGURE 14:**
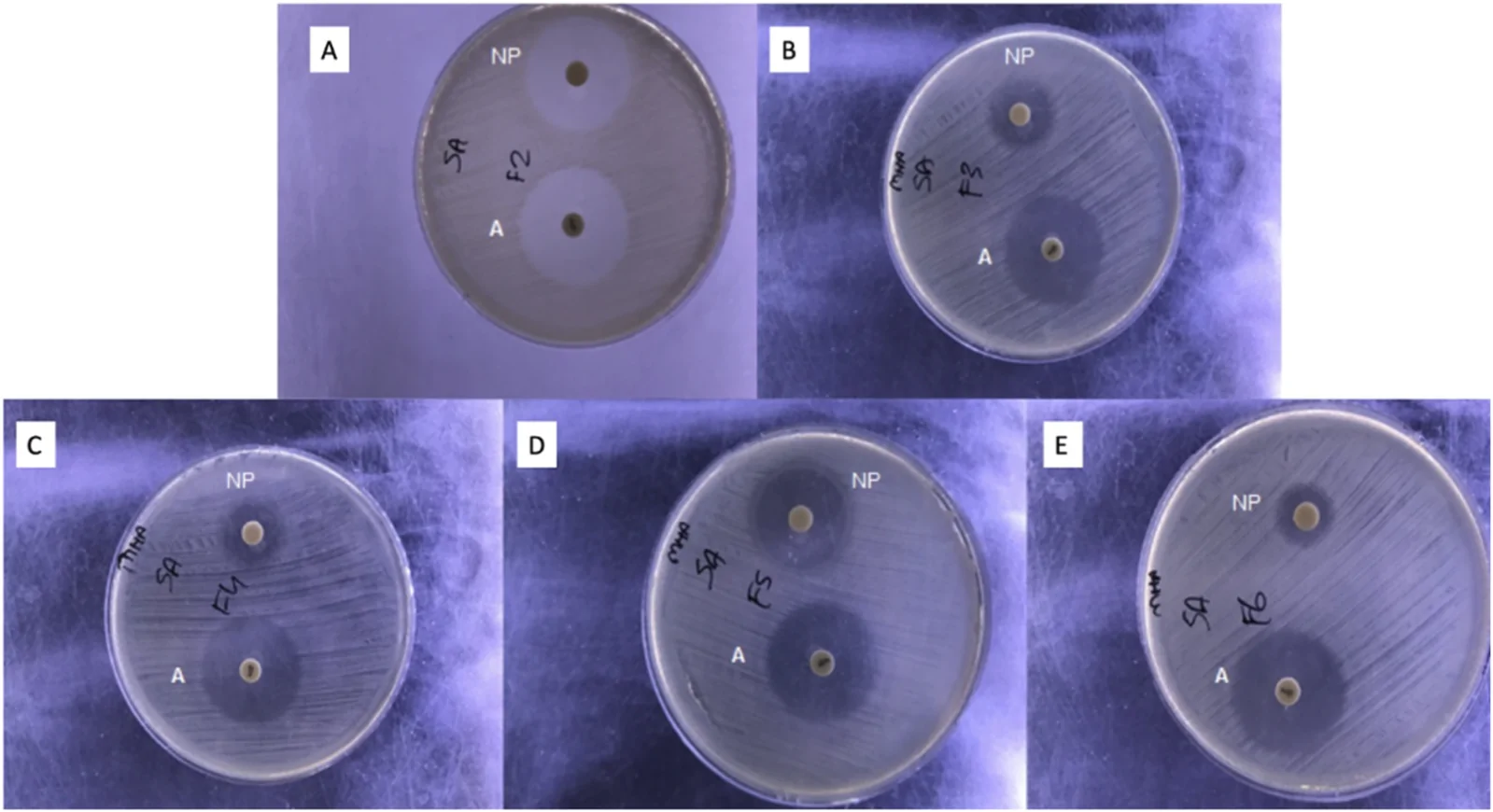
Zone diameter images of formulations and antibiotics applied on *Staphylococcus aureus*. **(A)** F2, **(B)** F3, **(C)** F4, **(D)** F5, **(E)** F6 formulations). NP; formulation, A; antibiotic, SA; It refers to *Staphylococcus aureus*. Antibacterial activity was assessed based on the diameter of the inhibition zones formed at the sites of bacterial growth suppression. Each formulation was compared to erythromycin as a reference antibiotic against *Staphylococcus aureus* and *Escherichia coli*.

**FIGURE 15:**
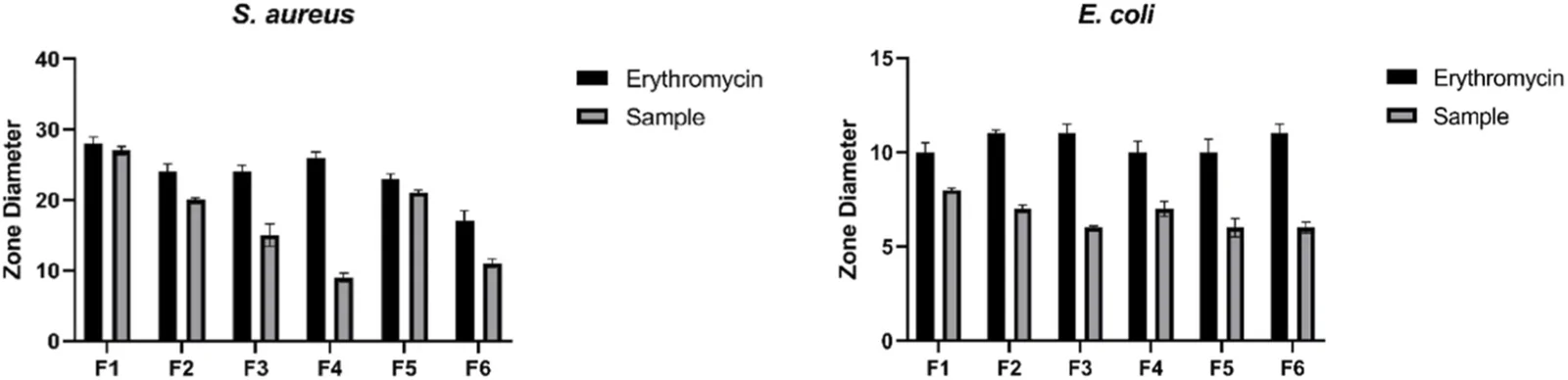
Statistical analysis of zone diameter of formulations against *Staphylococcus aureus* and *Escherichia coli*. Each formulation was compared with erythromycin. (Sample: produced nanofiber formulation). (Results of the two-way ANOVA comparing the formulations to one another for *Staphylococcus aureus* and *Escherichia coli* growth suppression).

The developed nanofibrous wound dressing, incorporating *C. coggygria* extract and HA within a PCL matrix, presents a novel approach not previously reported in the literature. While direct comparisons with existing dressings are limited due to the unique composition of our formulation, we can evaluate its potential advantages relative to current wound dressing systems. Previous studies have demonstrated the efficacy of HA nanofiber wound dressings in promoting wound healing. For instance, a study reported that sterilized HA nanofiber dressings exhibited superior healing outcomes compared to traditional dressings like gauze with vaseline, adhesive bandages, and antibiotic dressings. These benefits are attributed to HA’s ability to maintain a moist wound environment and facilitate cell migration. Research involving polylactide (PLA) and chitosan-based nanofiber dressings has shown that PLA-based dressings can accelerate the proliferative and re-epithelialization phases of wound healing, while chitosan-based dressings enhance the inflammatory phase. These findings highlight the importance of selecting appropriate polymer matrices to modulate specific healing phases. The incorporation of natural extracts into nanofiber dressings has been explored to enhance antimicrobial and antioxidant properties. For example, nanofiber dressings containing peppermint extract demonstrated significant antibacterial activity and improved wound healing outcomes. However, the combination of *C. coggygria* extract with HA in a PCL nanofiber matrix remains unexplored.

The integration of *C. coggygria* extract recognized for its antimicrobial and anti-inflammatory properties with HA to promote cell proliferation and migration combines components with complementary functions: PCL provides mechanical integrity, HA hydrophilicity and biological compatibility, and the extract antibacterial activity. Whether these components act additively, synergistically or antagonistically was not determined, as no formal interaction analysis was performed. Furthermore, the PCL matrix provides structural integrity and mechanical strength essential for scaffold durability and practical application. Electrospinning also facilitates the uniform distribution of bioactive compounds within the nanofibers at the wound site. While these developed dressings show substantial promise, further *in vivo* studies and clinical trials are required to fully evaluate their efficacy and safety relative to conventional wound management systems. Future investigations should focus on optimizing extract and HA concentrations, assessing performance across diverse wound models, and exploring the feasibility of scale-up and commercial translation.

Limitations: Several limitations of this study should be stated explicitly. First, the six formulations differ in solvent system and in two process parameters as well as in composition, so the observed differences reflect the fabrication condition as a whole and not composition alone. Second, all biological testing was performed on extracts of the mats in culture medium rather than on intact mats in contact with cells and therefore does not reproduce the geometry of the intended application. Third, scratch closure reflects both migration and proliferation, and no proliferation control was applied; the data are reported as closure and are not evidence of a specific effect on migration. Fourth, the largest inhibition zone in the antibacterial assay was produced by the mat containing neither HA nor extract, which means that the absolute zone diameters cannot be taken as a measure of the antibacterial contribution of the extract. Fifth, no *in vivo* model was used, and the endpoints measured here are *in vitro* surrogates that are not equivalent to wound healing. Sixth, the effects of sterilization on the mats, and their stability on storage, were not assessed. Seventh, no release study was performed. The rate and mechanism by which the polyphenolic constituents leave the PCL/HA matrix were not determined, and the present data must not be interpreted as evidence of controlled or sustained release. Eighth, the actual extract content and loading efficiency of the electrospun fibers were not determined; the extract content is reported only as the theoretical loading calculated from the formulation composition, and the biological results cannot be normalized to a measured extract dose. Ninth, no extract-only control was included in the biological assays, so the specific contribution of the *C. coggygria* extract to cytocompatibility, scratch closure and antibacterial activity cannot be established from these data. Tenth, residual solvent was not quantified: no GC or GC–MS determination of residual DMF or acetone was performed, the post-production drying procedure was, and no solvent-only control disk or vehicle control was included. The origin of the unexpectedly high antibacterial activity of F1 therefore cannot be determined, and it is not interpreted as antibacterial activity of the polymer. Eleventh, the cytotoxicity assay was not fully compliant with ISO 10993-5: extraction was performed at 1 mg/mL, below the mass-to-volume ratio specified for irregularly shaped materials, and no material or vehicle control was included; the results are therefore interpreted as an indicator of cytotoxicity rather than as a formal biocompatibility assessment. Finally, the extract is a commercial standardized product. Although the batch used was fully specified by the manufacturer and independently verified for total polyphenols and flavonoids, a complete chromatographic fingerprint of all constituents was not obtained, and batch-to-batch variability cannot be excluded. Each of these points defines a specific objective for the continuation of this work.

## 4 Conclusion

Electrospun PCL nanofibers containing HA and a standardized *C. coggygria* leaf extract were fabricated and characterized *in vitro*. All six compositions could be electrospun into continuous submicron fibers (390–635 nm), although the PCL-only fiber (F1) contained numerous beads. Surface wettability was governed by the extract content rather than by HA. F5 (10% PCL, 0.5% HA, 1% extract) gave the highest tensile strength (2.40 ± 0.15 MPa) together with the highest elongation at break (178% ± 18%). In L929 fibroblasts at 24 h, F5 was the only composition that maintained viability above 80% across the whole tested range of 0.062–1 mg/mL, whereas F4 (3% extract without HA) was clearly cytotoxic and produced the highest apoptotic and necrotic indices at 48 h. All compositions increased scratch closure relative to the untreated control at 24 h, and all produced larger inhibition zones against *S. aureus* than against *E. coli*. No release study was performed in this work. Consequently, no conclusions can be drawn regarding the rate or mechanism of extract release from the fibers, and the available data are not considered evidence of controlled or sustained release. Establishing the release profile of the polyphenolic constituents from the PCL/HA matrix, and fitting it to the standard kinetic models, is the first objective of the follow-up study. It must be emphasized that the six formulations differ not only in composition but also in the solvent system (two-solvent for the HA-free fibers, three-solvent for the HA-containing fibers) and in two process parameters (flow rate and needle–collector distance, both of which had to be changed for the extract-containing solutions in order to obtain a stable jet). The comparisons that follow are therefore comparisons between whole fabrication conditions and not between compositions alone, and differences in fiber diameter, bead density and mechanical properties cannot be attributed exclusively to HA or to the extract.

On this basis, F5 is proposed as the lead composition for further development, and the demonstration of therapeutic efficacy will require *in vivo* wound models, which together with sterilization and stability studies constitute the next stage of this work. In the present study, electrospun PCL nanofibrous wound dressings successfully integrated HA and C. coggygria extract. Comprehensive *in vitro* evaluations including cell viability, apoptosis/necrotic indexing, scratch wound assays, and antibiogram testing demonstrated that the addition of HA and optimized levels of C. coggygria extract significantly enhanced cell proliferation, migration, and antibacterial activity against *S. aureus*. However, these biological improvements were found to be strictly dose-dependent, with higher extract concentrations leading to increased cytotoxicity. Consequently, formulation F5 (comprising PCL, 0.5% HA, and 1% extract) exhibited the most favorable and balanced profile for effective wound management. Future work will focus on evaluating sterilization impacts, stability, and *in vivo* animal models to confirm clinical translation and therapeutic efficacy.

## Data Availability

The original contributions presented in the study are included in the article/Supplementary Material, further inquiries can be directed to the corresponding authors.

## References

[B1] Adamu B. F. Jing G. Xiangnan Y. Shaojie T. (2024). Indigenous Ethiopian traditional medicinal plant leaf extract, *Croton macrostachyus*, loaded PCL electrospun nanofibrous mat as potential wound dressing: *in vitro* analysis. J. Bioact. Compat. Polym. 39 (4), 236–250. 10.1177/08839115241256950

[B4] Aksoy H. Sen A. Sancar M. Sekerler T. Akakin D. Bitis L. (2016). Ethanol extract of *Cotinus coggygria* leaves accelerates wound healing process in diabetic rats. Pharm. Biol. 54 (11), 2732–2736. 10.1080/13880209.2016.1181660 27180800

[B5] Antal D. S. Ardelean F. Jijie R. Pinzaru I. Soica C. Dehelean C. (2021). Integrating ethnobotany, phytochemistry, and pharmacology of *Cotinus coggyg*ria and *toxicodendron vernicifluum*: what predictions can be made for the European smoke tree? Front. Pharmacol. 12, 662852. 10.3389/fphar.2021.662852 PMC8092975 33953688

[B6] Ascione F. Vasaturo A. Caserta S. D'Esposito V. Formisano P. Guido S. (2016). Comparison between fibroblast wound healing and cell random migration assays *in vitro* . Exp. Cell Res. 347 (1), 123–132. 10.1016/j.yexcr.2016.07.015 27475838

[B7] Astaneh M. E. Fereydouni N. (2023). A focused review on hyaluronic acid contained nanofiber formulations for diabetic wound healing. Int. J. Biol. Macromol. 253 (Pt 8), 127607. 10.1016/j.ijbiomac.2023.127607 37871723

[B8] Batool S. Ali Z. Alamri A. H. Al Fatease A. Lahiq A. A. Asiri A. (2025). Development and statistical optimization of camptothecin loaded hyaluronic acid and zein polymeric nanoparticles towards the treatment of melanoma. Int. J. Biol. Macromol. 321, 146330. 10.1016/j.ijbiomac.2025.146330 40716545

[B9] Baytop T. (1999). Therapy with Plants in Turkey (Past and Present). Istanbul: Nobel Medical Bookhouse.

[B10] Bozkaya E. Türk M. Ekici H. Karahan S. (2023). Investigation of the biocompatibility and *in vivo* wound healing effect of *cotinus coggyria* extracts. Ank Univ. Vet. Fak. Derg. 71 (3), 269–280. 10.33988/auvfd.1217177

[B20] De Ieso M. L. Pei J. V. (2018). An accurate and cost-effective alternative method for measuring cell migration with the circular wound closure assay. Biosci. Rep. 38 (5). 10.1042/BSR20180698 30232234 PMC6209583

[B36] Della Sala F. Longobardo G. Fabozzi A. di Gennaro M. Borzacchiello A. (2022). Hyaluronic acid-based wound dressing with antimicrobial properties for wound healing application. Appl. Sci. 12 (6), 3091. 10.3390/app12063091

[B11] Deniz F. S. S. Salmas R. E. Emerce E. Tatli Cankaya I. I. Yusufoglu H. S. Orhan I. E. (2020). Evaluation of collagenase, elastase and tyrosinase inhibitory activities of *Cotinus coggygria* scop. through *in vitro* and *in silico* approaches. South Afr. J. Bot. 132, 277–288. 10.1016/j.sajb.2020.05.017

[B12] Eskitoros-Togay Ş. M. Bulbul Y. E. Tort S. Korkmaz F. D. Acartürk F. Dilsiz N. (2019). Fabrication of doxycycline-loaded electrospun PCL/PEO membranes for a potential drug delivery system. Int. J. Pharm. 565, 83–94. 10.1016/j.ijpharm.2019.04.073 31063838

[B13] Fadaie M. Mirzaei E. Geramizadeh B. Asvar Z. (2018). Incorporation of nanofibrillated chitosan into electrospun PCL nanofibers makes scaffolds with enhanced mechanical and biological properties. Carbohydr. Polym. 199, 628–640. 10.1016/j.carbpol.2018.07.061 30143171

[B14] Fadil F. Affandi N. D. N. Misnon M. I. Bonnia N. N. Harun A. M. Alam M. K. (2021). Review on electrospun nanofiber-applied products. Polym. (Basel) 13 (13), 2087. 10.3390/polym13132087 PMC8271930 34202857

[B15] Fahimirad S. Abtahi H. Satei P. Ghaznavi-Rad E. Moslehi M. Ganji A. (2021). Wound healing performance of PCL/chitosan based electrospun nanofiber electrosprayed with curcumin loaded chitosan nanoparticles. Carbohydr. Polym. 259, 117640. 10.1016/j.carbpol.2021.117640 33673981

[B16] Fronza M. Heinzmann B. Hamburger M. Laufer S. Merfort I. (2009). Determination of the wound healing effect of *calendula* extracts using the scratch assay with 3T3 fibroblasts. J. Ethnopharmacol. 126 (3), 463–467. 10.1016/j.jep.2009.09.014 19781615

[B17] Gedik B. Erdem M. A. (2025). Electrospun PCL membranes for localized drug delivery and bone regeneration. BMC Biotechnol. 25 (1), 31. 10.1186/s12896-025-00965-7 PMC12023437 40275261

[B18] Huang W. Jin Z. Chen Y. Yang G. Xu Y. (2025). Preparation, characterization, and drug release of electrospun core‐shell structured hyaluronic acid‐bovine serum albumin/poly (ε‐caprolactone) nanofiber membranes. J. Appl. Polym. Sci. 142, e56794. 10.1002/app.56794

[B19] Huerta-Ángeles G. Mixcoha E. (2025). Recent advances, research trends, and clinical relevance of hyaluronic acid applied to wound healing and regeneration. Appl. Sci. 15 (2), 536. 10.3390/app15020536

[B21] Ilbasmis-Tamer S. Saral-Acarca E. S. Tort S. Yücel Ç. Tamer U. Acartürk F. (2022). Fabrication and characterization of starch-copper nanoparticles/rutin nanofiber hybrid scaffold. J. Drug Deliv. Sci. Technol. 72, 103401. 10.1016/j.jddst.2022.103401

[B22] Inal M. Mulazimoglu G. (2019). Production and characterization of bactericidal wound dressing material based on gelatin nanofiber. Int. J. Biol. Macromol. 137, 392–404. 10.1016/j.ijbiomac.2019.06.119 31233795

[B23] Ito T. Koyama Y. Otsuka M. (2010). Analysis of the surface structure of DNA/polycation/hyaluronic acid ternary complex by raman microscopy. J. Pharm. Biomed. Anal. 51 (1), 268–272. 10.1016/j.jpba.2009.07.024 19692196

[B24] Jia J. Lin Z. Zhu J. Liu Y. Hu Y. Fang K. (2024). Anti-adhesive and antibacterial chitosan/PEO nanofiber dressings with high breathability for promoting wound healing. Int. J. Biol. Macromol. 261 (Pt 1), 129668. 10.1016/j.ijbiomac.2024.129668 38278380

[B25] Khalid M. Adnan M. Farooq M. Yoshinori Y. Park J. Ullah A. (2025). Efficient water-soluble Cu (II) complex-immobilized electrospun hydrophobic polycaprolactone nanofiber composites for highly controlled and long-term release. ACS Omega 10 (13), 12961–12971. 10.1021/acsomega.4c09305 40224431 PMC11983174

[B26] Khan A. U. R. Huang K. Khalaji M. S. Yu F. Xie X. Zhu T. (2021). Multifunctional bioactive core-shell electrospun membrane capable to terminate inflammatory cycle and promote angiogenesis in diabetic wound. Bioact. Mater 6 (9), 2783–2800. 10.1016/j.bioactmat.2021.01.040 PMC7900605 33665509

[B27] Khunova V. Kovacova M. Olejnikova P. Ondreas F. Spitalsky Z. Ghosal K. (2022). Antibacterial electrospun polycaprolactone nanofibers reinforced by halloysite nanotubes for tissue engineering. Polym. (Basel) 14 (4), 746. 10.3390/polym14040746 PMC8876556 35215658

[B28] Kultur S. (2007). Medicinal plants used in kirklareli province (Turkey). J. Ethnopharmacol. 111 (2), 341–364. 10.1016/j.jep.2006.11.035 17257791

[B29] Kumelachew D. M. Li C. Zhang Y. Adamu B. F. Zhao F. Wang F. J. (2025). Calcium carbonate modified polyurethane shell in polycaprolactone based core-shell fibre integrated 5-fluorouracil delivery for colon treatment: *in vitro* characterization. J. Polym. Res. 32 (10), 336. 10.1007/s10965-025-04564-0

[B30] Lababidi J. M. Fytory M. El-Qassas J. Gad A. T. Mohamed O. El-Badri N. (2025). Promising wound healing activity of *saussurea costus* loaded PCL-gelatin nanofibers. Mater Adv. 6 (10), 3114–3131. 10.1039/D4MA01196A

[B45] La Verde G. Sasso A. Rusciano G. Capaccio A. Fusco S. Mayol L. (2022). Characterization of hyaluronic acid-coated PLGA nanoparticles by surface-enhanced raman spectroscopy. Int. J. Mol. Sci. 24 (1), 601. 10.3390/ijms24010601 36614044 PMC9820697

[B31] Mamedov N. Gardner Z. Craker L. E. (2005). Medicinal plants used in Russia and central Asia for the treatment of selected skin conditions. J. Herbs Spices Med. Plants 11 (1-2), 191–222. 10.1300/J044v11n01_07

[B32] Matic S. Stanic S. Mihailovic M. Bogojevic D. (2016). *Cotinus coggygria* scop.: an overview of its chemical constituents, pharmacological and toxicological potential. Saudi J. Biol. Sci. 23 (4), 452–461. 10.1016/j.sjbs.2015.05.012 27298577 PMC4890191

[B33] Meng Q. Xu H. Li Y. Liu F. Shao H. Ling P. (2024). Conjugated electrospinning toward a polycaprolactone scaffold simultaneously containing micro-/nano-fibers for potential biomedical application. J. Polym. Res. 31, 301. 10.1007/s10965-024-04152-8

[B34] Rahman S. Jan G. Jan F. G. Rahim H. U. (2022). Phytochemical investigation and therapeutical potential of *Cotinus coggygria* scop. in alloxan‐induced diabetic mice. Oxid. Med. Cell Longev. 2022 (1), 8802178. 10.1155/2022/8802178 36624877 PMC9825219

[B35] Rahman M. Kabir M. Li K. Li Y. Chen S. Wu S. (2024). Electrospun zeolitic imidazole framework-8 loaded silk fibroin/polycaprolactone nanofibrous scaffolds for biomedical application. J. Mech. Behav. Biomed. Mater 160, 106769. 10.1016/j.jmbbm.2024.106769 39418744

[B37] Samavati S. S. Kashanian S. Derakhshankhah H. Abuzade R. A. Sajadimajd S. Rabiei M. (2023). PCL and PCL-jaft nanofiber, synthesis, characterization, and histological comparison of the quality of wound healing. J. Drug Deliv. SciTechnol 84, 104457. 10.1016/j.jddst.2023.104457

[B38] Sen A. Ertas B. Cevik O. Yildirim A. Kayali D. G. Akakin D. (2023). *Cotinus coggygria* scop. attenuates acetic acid-induced colitis in rats by regulation of inflammatory mediators. Appl. Biochem. Biotechnol. 195 (11), 7021–7036. 10.1007/s12010-023-04474-1 36976506

[B39] Shafiei S. S. Shavandi M. Ahangari G. Shokrolahi F. (2016). Electrospun layered double hydroxide/poly (ε-caprolactone) nanocomposite scaffolds for adipogenic differentiation of adipose-derived mesenchymal stem cells. Appl. Clay Sci. 127-128, 52–63. 10.1016/j.clay.2016.04.004

[B40] Sivakumar S. Sadaiyandi V. Swaminathan S. Ramalingam R. (2024). Biocompatibility, anti-hemolytic, and antibacterial assessments of electrospun PCL/collagen composite nanofibers loaded with *acanthophora spicifera* extracts mediated copper oxide nanoparticles. Biocatal. Agric. Biotechnol. 55, 102983. 10.1016/j.bcab.2023.102983

[B41] Smith G. P. McLaughlin A. W. Clarkson A. N. Gordon K. C. Walker G. F. (2017). Raman microscopic imaging of electrospun fibers made from a polycaprolactone and polyethylene oxide blend. Vib. Spectr. 92, 27–34. 10.1016/j.vibspec.2017.05.002

[B42] Tort S. Acarturk F. Besikci A. (2017). Evaluation of three-layered doxycycline-collagen loaded nanofiber wound dressing. Int. J. Pharm. 529 (1-2), 642–653. 10.1016/j.ijpharm.2017.07.027 28705624

[B43] Tsanaktsidou E. Kammona O. Kiparissides C. (2019). On the synthesis and characterization of biofunctional hyaluronic acid based injectable hydrogels for the repair of cartilage lesions. Eur. Polym. J. 114, 47–56. 10.1016/j.eurpolymj.2019.02.024

[B44] Uppal R. Ramaswamy G. N. Arnold C. Goodband R. Wang Y. (2011). Hyaluronic acid nanofiber wound dressing--production, characterization, and *in vivo* behavior. J. Biomed. Mater Res. B Appl. Biomater. 97 (1), 20–29. 10.1002/jbm.b.31776 21290571

