# Supplementary material for: Cotinus coggygria Scop. extract-loaded polycaprolactone/hyaluronic acid nanofibers as candidate wound dressings: in vitro characterization, cytocompatibility and antibacterial evaluation
Source: Front Pharmacol. 2026 Oct 23;17:1948812. doi: 10.3389/fphar.2026.1948812 (PMC13739705; doi:10.3389/fphar.2026.1948812)
Supplement: Supplementary file 1 [file DataSheet1.pdf]

Supplementary material for

***Cotinus coggygria* Scop. Extract-Loaded Nanofiber Wound Dressings for Accelerated Wound Healing**

Eylul Su Saral-Acarca[1,2], Serdar Tort[1], Mustafa Türk[3], Fatma Sezer Senol Deniz[4], Ilkay Erdoğan Orhan*[5], Sibel Ilbasmis-Tamer[*1]

1 Faculty of Pharmacy, Department of Pharmaceutical Technology, Gazi University, Ankara, Türkiye
2 Faculty of Pharmacy, Department of Pharmaceutical Technology, Başkent University, Ankara, Türkiye
3 Faculty of Engineering, Department of Bioengineering, Kırıkkale University, Kırıkkale, Türkiye
4 Faculty of Pharmacy, Department of Pharmacognosy, Gazi University, Ankara, Türkiye
5 Faculty of Pharmacy, Department of Pharmacognosy, Lokman Hekim University, Ankara, Türkiye

Corresponding Authors

Prof. Sibel Ilbasmis-Tamer

Gazi University, Department of Pharmaceutical Technology, Ankara, Turkey, Orcid: 0000-0003-0361-7105;

e- mail: ilbasmis@gazi.edu.tr

Prof. Dr. Ilkay Erdoğan Orhan

Lokman Hekim University, Department of Pharmacognosy, Ankara, Turkey, Orcid: 0000-0002-7379-5436

e- mail: ilkay.erdoganorhan@lokmanhekim.edu.tr

2. Methods

2.3 Characterizations of Electrospun Nanofibers

2.3.1 Contact Angle Study

The contact angle measurements were performed by Attension Theta Lite optical tensiometer and measured by the sessile drop technique. The nanofibers were placed on a sample holder. Distilled water was used to form the drops and the drop volume was 5 µL for each formulation and contact angle values were measured. Three different measurements were carried out.

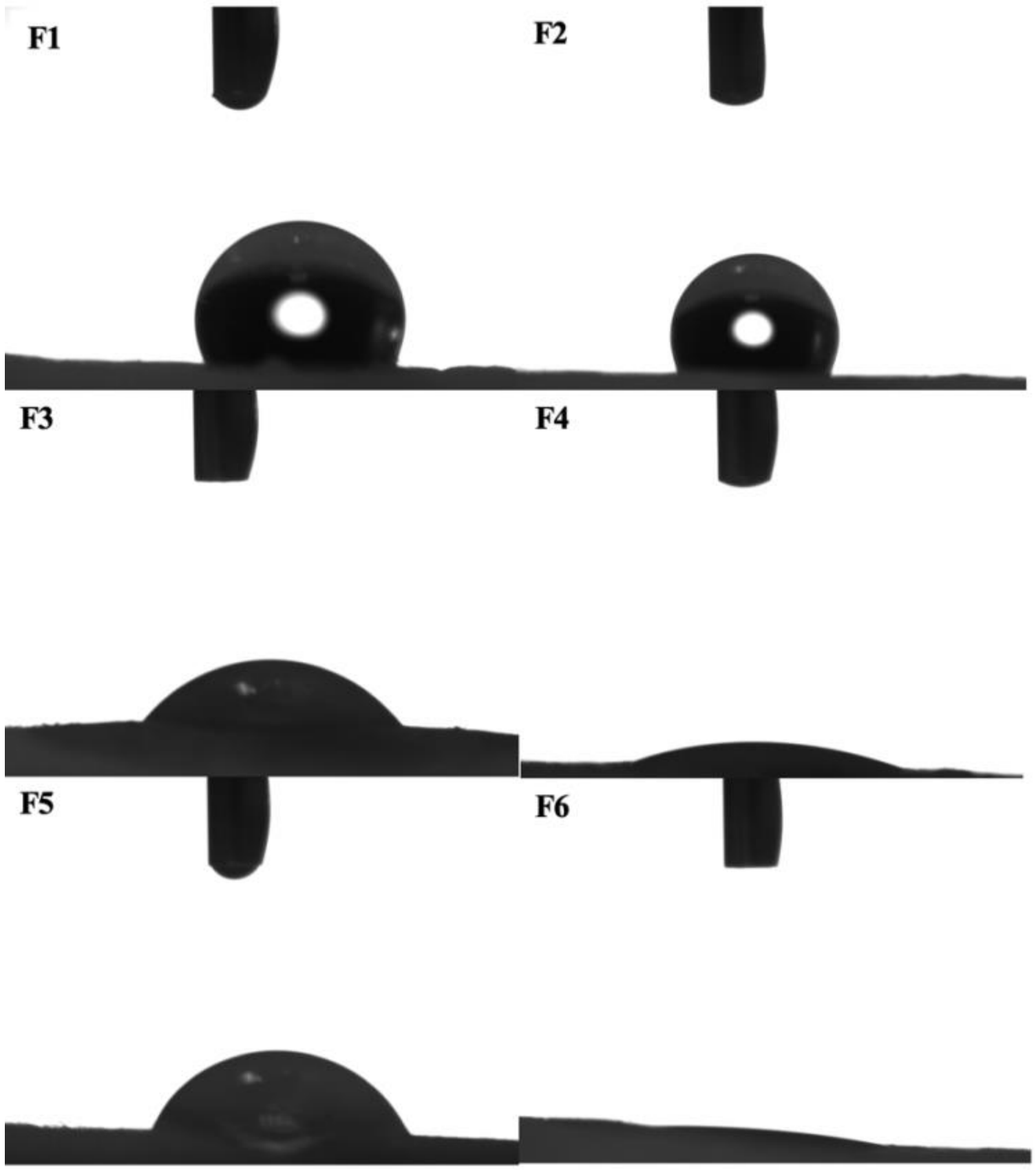

Figure 1. Actual images of contact angle studies. (F1: produced only with PCL, F2: produced with PCL and HA, F3 and F4: produced with PCL and *Cotinus cogyggria* extract at different concentrations, F5 and F6: produced with PCL, HA and *Cotinus cogyggria* extract at different concentrations.)

2.3.2 Mechanical Properties

The mechanical properties of nanofibers were determined with a texture analyzer (Stable Micro Systems, TA.XT. PlusTexture Analyzer, UK). Three samples of each formulation were cut into 1 cm × 3 cm size samples and placed. As a result of the study, elongation at break and tensile strength values were measured. Three different measurements were carried out.

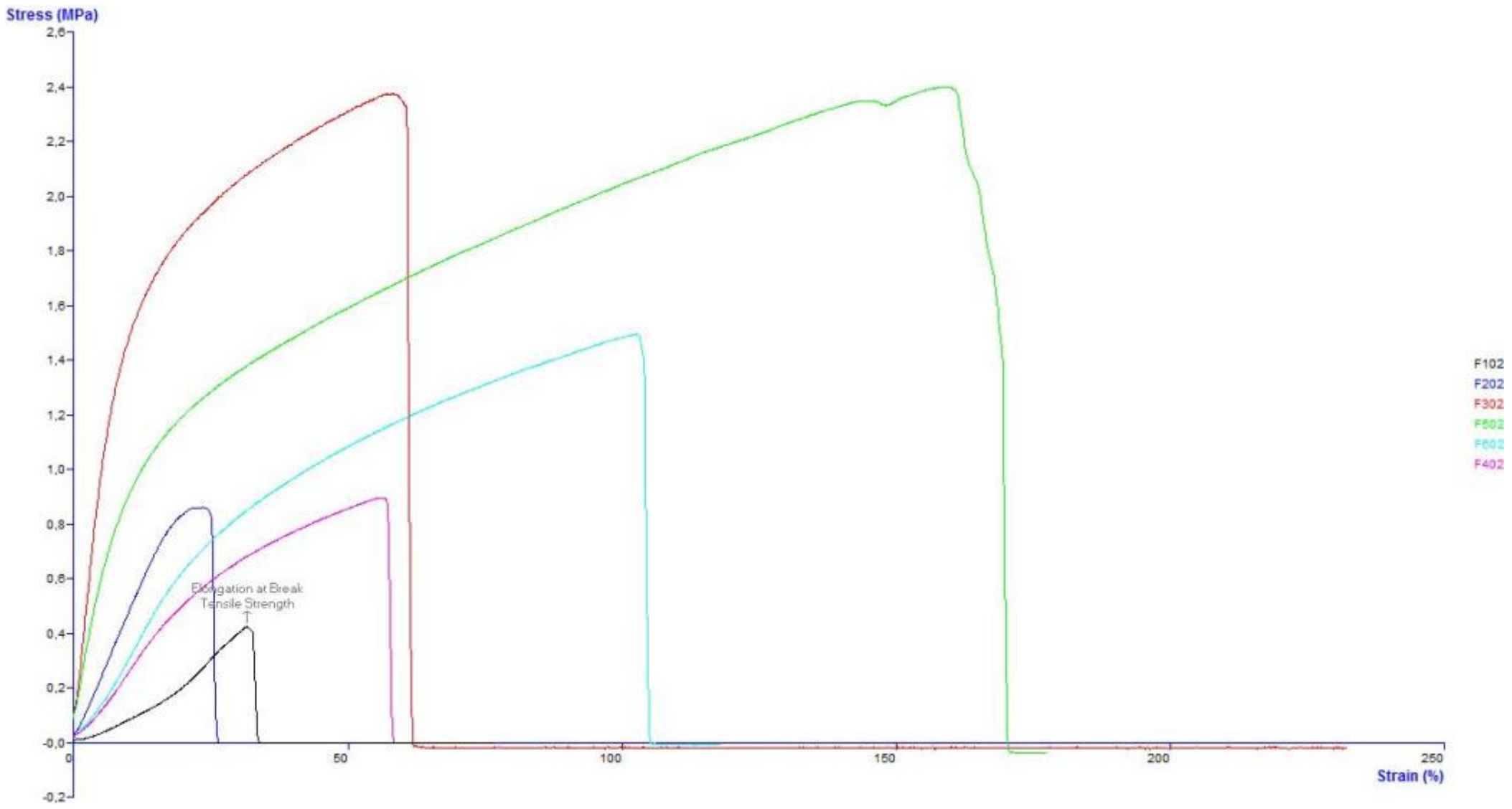

Figure 2. Tensile curves from mechanical characterization of each formulation.

2.3.6 Statistical analysis with details

For %cell viability graph (Figure 5) statistical analysis dots could not be added to show the significance of the values due to the large amount of data and the inability to fit the visual. However, the raw data of the statistical study is presented below.

1 mg/mL	Summary	Adjusted P Value
Negative Control vs. Positive Control	****	<0.0001
Negative Control vs. F1	****	<0.0001
Negative Control vs. F2	****	<0.0001
Negative Control vs. F3	****	<0.0001
Negative Control vs. F4	****	<0.0001
Negative Control vs. F5	****	<0.0001
Negative Control vs. F6	****	<0.0001
Positive Control vs. F1	****	<0.0001
Positive Control vs. F2	****	<0.0001
Positive Control vs. F3	****	<0.0001
Positive Control vs. F4	ns	0.8091
Positive Control vs. F5	****	<0.0001
Positive Control vs. F6	****	<0.0001
F1 vs. F2	****	<0.0001
F1 vs. F3	****	<0.0001
F1 vs. F4	****	<0.0001
F1 vs. F5	****	<0.0001

F1 vs. F6	****	<0.0001
F2 vs. F3	****	<0.0001
F2 vs. F4	****	<0.0001
F2 vs. F5	****	<0.0001
F2 vs. F6	***	0.0005
F3 vs. F4	****	<0.0001
F3 vs. F5	****	<0.0001
F3 vs. F6	****	<0.0001
F4 vs. F5	****	<0.0001
F4 vs. F6	****	<0.0001
F5 vs. F6	****	<0.0001
0.5 mg/mL		
Negative Control vs. Positive Control	****	<0.0001
Negative Control vs. F1	****	<0.0001
Negative Control vs. F2	****	<0.0001
Negative Control vs. F3	****	<0.0001
Negative Control vs. F4	****	<0.0001
Negative Control vs. F5	****	<0.0001
Negative Control vs. F6	****	<0.0001
Positive Control vs. F1	****	<0.0001
Positive Control vs. F2	****	<0.0001
Positive Control vs. F3	****	<0.0001
Positive Control vs. F4	****	<0.0001
Positive Control vs. F5	****	<0.0001
Positive Control vs. F6	****	<0.0001
F1 vs. F2	****	<0.0001
F1 vs. F3	****	<0.0001
F1 vs. F4	****	<0.0001
F1 vs. F5	****	<0.0001
F1 vs. F6	ns	0.5920
F2 vs. F3	****	<0.0001
F2 vs. F4	****	<0.0001
F2 vs. F5	****	<0.0001

F2 vs. F6	****	<0.0001
F3 vs. F4	****	<0.0001
F3 vs. F5	****	<0.0001
F3 vs. F6	****	<0.0001
F4 vs. F5	****	<0.0001
F4 vs. F6	****	<0.0001
F5 vs. F6	****	<0.0001
0.25 mg/mL		
Negative Control vs. Positive Control	****	<0.0001
Negative Control vs. F1	****	<0.0001
Negative Control vs. F2	****	<0.0001
Negative Control vs. F3	****	<0.0001
Negative Control vs. F4	****	<0.0001
Negative Control vs. F5	ns	0.6849
Negative Control vs. F6	****	<0.0001
Positive Control vs. F1	****	<0.0001
Positive Control vs. F2	****	<0.0001
Positive Control vs. F3	****	<0.0001
Positive Control vs. F4	****	<0.0001
Positive Control vs. F5	****	<0.0001
Positive Control vs. F6	****	<0.0001
F1 vs. F2	****	<0.0001
F1 vs. F3	****	<0.0001
F1 vs. F4	*	0.0423
F1 vs. F5	****	<0.0001
F1 vs. F6	***	0.0005
F2 vs. F3	****	<0.0001
F2 vs. F4	****	<0.0001
F2 vs. F5	****	<0.0001
F2 vs. F6	ns	0.9920
F3 vs. F4	****	<0.0001
F3 vs. F5	****	<0.0001
F3 vs. F6	****	<0.0001

F4 vs. F5	****	<0.0001
F4 vs. F6	****	<0.0001
F5 vs. F6	****	<0.0001
0.125 mg/mL		
Negative Control vs. Positive Control	****	<0.0001
Negative Control vs. F1	****	<0.0001
Negative Control vs. F2	****	<0.0001
Negative Control vs. F3	****	<0.0001
Negative Control vs. F4	****	<0.0001
Negative Control vs. F5	**	0.0034
Negative Control vs. F6	****	<0.0001
Positive Control vs. F1	****	<0.0001
Positive Control vs. F2	****	<0.0001
Positive Control vs. F3	****	<0.0001
Positive Control vs. F4	****	<0.0001
Positive Control vs. F5	****	<0.0001
Positive Control vs. F6	****	<0.0001
F1 vs. F2	****	<0.0001
F1 vs. F3	****	<0.0001
F1 vs. F4	*	0.0138
F1 vs. F5	****	<0.0001
F1 vs. F6	****	<0.0001
F2 vs. F3	****	<0.0001
F2 vs. F4	**	0.0018
F2 vs. F5	****	<0.0001
F2 vs. F6	****	<0.0001
F3 vs. F4	****	<0.0001
F3 vs. F5	****	<0.0001
F3 vs. F6	ns	0.9648
F4 vs. F5	****	<0.0001
F4 vs. F6	****	<0.0001
F5 vs. F6	****	<0.0001
0.062 mg/mL		

Negative Control vs. Positive Control	****	<0.0001
Negative Control vs. F1	****	<0.0001
Negative Control vs. F2	****	<0.0001
Negative Control vs. F3	*	0.0142
Negative Control vs. F4	ns	0.0883
Negative Control vs. F5	****	<0.0001
Negative Control vs. F6	****	<0.0001
Positive Control vs. F1	****	<0.0001
Positive Control vs. F2	****	<0.0001
Positive Control vs. F3	****	<0.0001
Positive Control vs. F4	****	<0.0001
Positive Control vs. F5	****	<0.0001
Positive Control vs. F6	****	<0.0001
F1 vs. F2	****	<0.0001
F1 vs. F3	****	<0.0001
F1 vs. F4	****	<0.0001
F1 vs. F5	****	<0.0001
F1 vs. F6	****	<0.0001
F2 vs. F3	****	<0.0001
F2 vs. F4	****	<0.0001
F2 vs. F5	****	<0.0001
F2 vs. F6	****	<0.0001
F3 vs. F4	ns	0.9985
F3 vs. F5	****	<0.0001
F3 vs. F6	****	<0.0001
F4 vs. F5	****	<0.0001
F4 vs. F6	****	<0.0001
F5 vs. F6	ns	0.9523

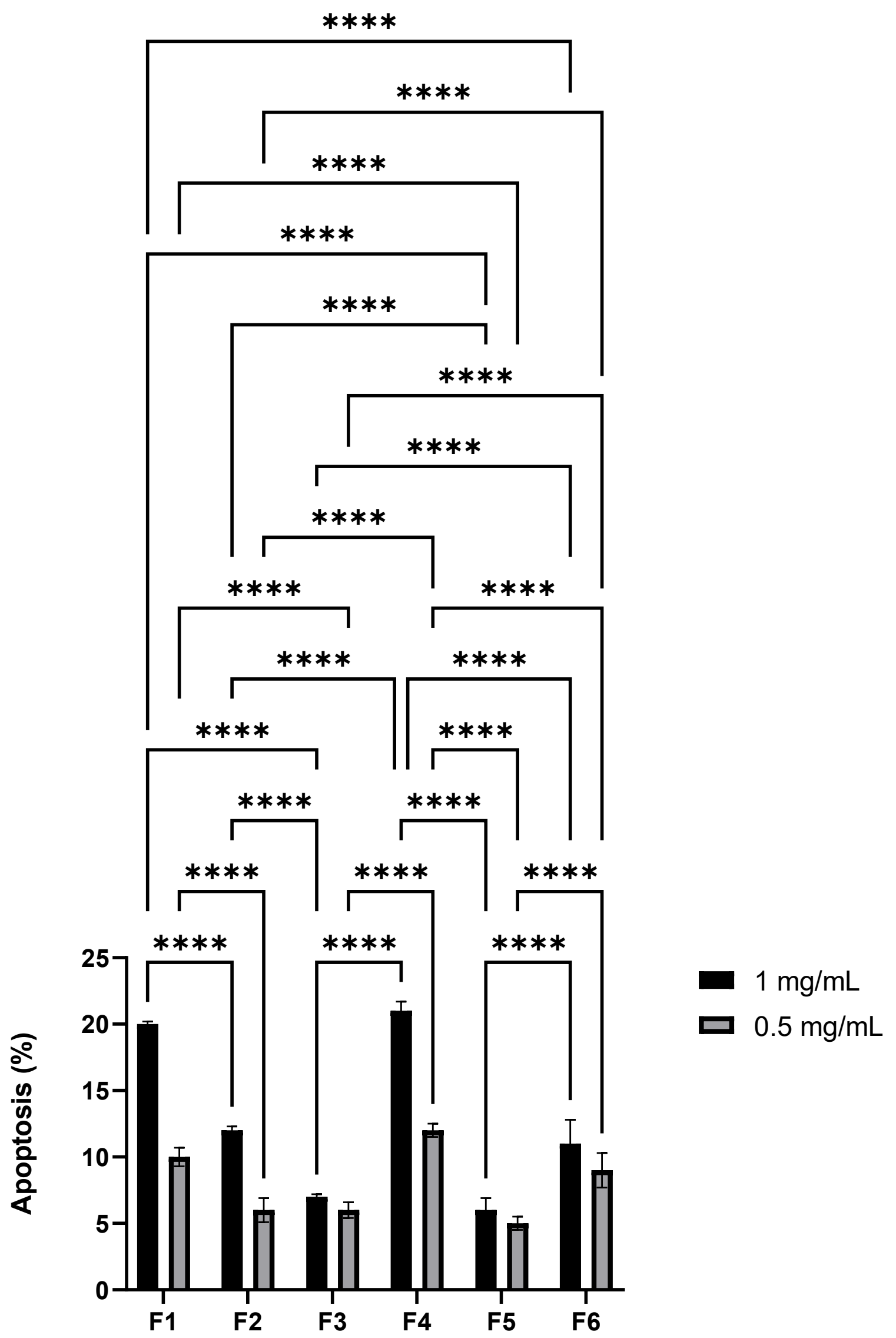

Figure 3. % Apoptosis graph with statistical significant values ($p < 0.0001$)

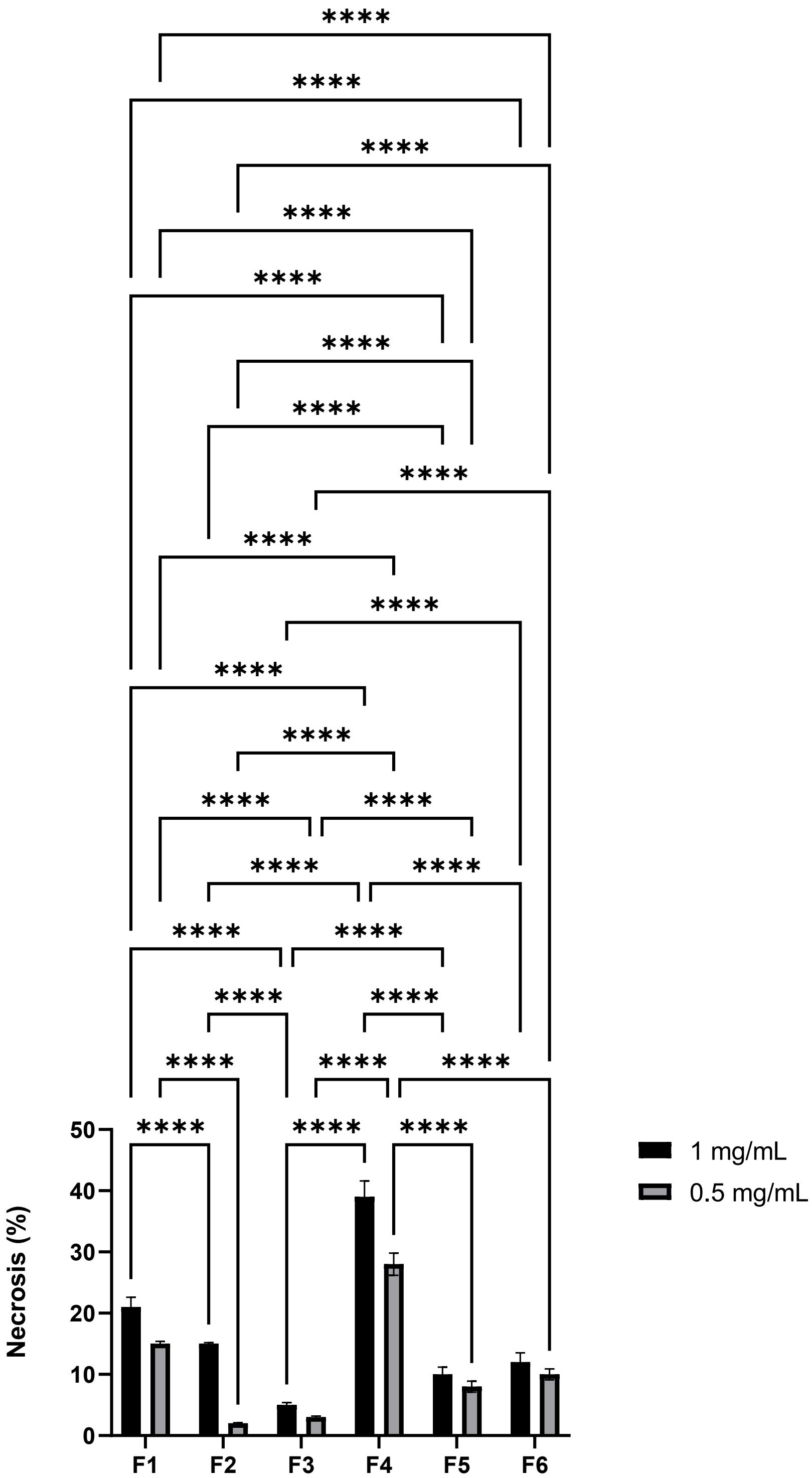

Figure 4. % Necrosis graph with statistical significant values ($p < 0.0001$)

For wound closure graph (Figure 10) statistical analysis dots could not be added to show the significance of the values due to the large amount of data and the inability to fit the visual. However, the raw data of the statistical study is presented below.

12 hours	Summary	Adjusted P Value
Control vs. F1	****	<0.0001
Control vs. F2	****	<0.0001
Control vs. F3	***	0.0003
Control vs. F4	****	<0.0001
Control vs. F5	****	<0.0001
Control vs. F6	****	<0.0001
F1 vs. F2	***	0.0005
F1 vs. F3	****	<0.0001
F1 vs. F4	****	<0.0001
F1 vs. F5	ns	0.8577
F1 vs. F6	****	<0.0001
F2 vs. F3	****	<0,0001
F2 vs. F4	****	<0.0001
F2 vs. F5	****	<0.0001
F2 vs. F6	****	<0.0001
F3 vs. F4	ns	0.9951
F3 vs. F5	****	<0.0001
F3 vs. F6	ns	0.4721
F4 vs. F5	****	<0.0001
F4 vs. F6	ns	0.8577
F5 vs. F6	***	0.0005
24 hours		
Control vs. F1	****	<0.0001
Control vs. F2	****	<0.0001
Control vs. F3	****	<0.0001
Control vs. F4	****	<0.0001
Control vs. F5	****	<0.0001

Control vs. F6	****	<0.0001
F1 vs. F2	****	<0.0001
F1 vs. F3	****	<0.0001
F1 vs. F4	****	<0.0001
F1 vs. F5	ns	0.9951
F1 vs. F6	****	<0.0001
F2 vs. F3	****	<0.0001
F2 vs. F4	****	<0.0001
F2 vs. F5	****	<0.0001
F2 vs. F6	****	<0.0001
F3 vs. F4	****	<0.0001
F3 vs. F5	****	<0.0001
F3 vs. F6	****	<0.0001
F4 vs. F5	****	<0.0001
F4 vs. F6	****	<0.0001
F5 vs. F6	****	<0.0001

For antibacterial activity graph (Figure 12) statistical analysis dots could not be added to show the significance of the values due to the large amount of data and the inability to fit the visual. However, the raw data of the statistical study is presented below.

For *S. aureus*	Summary	Adjusted P Value
F1:Erythromycin vs. F1:Sample	ns	0.7617
F1:Erythromycin vs. F2:Erythromycin	****	<0.0001
F1:Erythromycin vs. F2:Sample	****	<0.0001
F1:Erythromycin vs. F3:Erythromycin	****	<0.0001
F1:Erythromycin vs. F3:Sample	****	<0.0001
F1:Erythromycin vs. F4:Erythromycin	*	0.0174
F1:Erythromycin vs. F4:Sample	****	<0.0001
F1:Erythromycin vs. F5:Erythromycin	****	<0.0001
F1:Erythromycin vs. F5:Sample	****	<0.0001
F1:Erythromycin vs. F6:Erythromycin	****	<0.0001
F1:Erythromycin vs. F6:Sample	****	<0.0001
F1:Sample vs. F2:Erythromycin	****	<0.0001
F1:Sample vs. F2:Sample	****	<0.0001
F1:Sample vs. F3:Erythromycin	****	<0,0001
F1:Sample vs. F3:Sample	****	<0.0001
F1:Sample vs. F4:Erythromycin	ns	0.7617
F1:Sample vs. F4:Sample	****	<0.0001
F1:Sample vs. F5:Erythromycin	****	<0.0001
F1:Sample vs. F5:Sample	****	<0.0001
F1:Sample vs. F6:Erythromycin	****	<0.0001
F1:Sample vs. F6:Sample	****	<0.0001
F2:Erythromycin vs. F2:Sample	****	<0.0001
F2:Erythromycin vs. F3:Erythromycin	ns	>0.9999
F2:Erythromycin vs. F3:Sample	****	<0.0001
F2:Erythromycin vs. F4:Erythromycin	*	0.0174
F2:Erythromycin vs. F4:Sample	****	<0.0001
F2:Erythromycin vs. F5:Erythromycin	ns	0.7617

F2:Erythromycin vs. F5:Sample	****	<0.0001
F2:Erythromycin vs. F6:Erythromycin	****	<0.0001
F2:Erythromycin vs. F6:Sample	****	<0.0001
F2:Sample vs. F3:Erythromycin	****	<0.0001
F2:Sample vs. F3:Sample	****	<0.0001
F2:Sample vs. F4:Erythromycin	****	<0.0001
F2:Sample vs. F4:Sample	****	<0.0001
F2:Sample vs. F5:Erythromycin	****	<0.0001
F2:Sample vs. F5:Sample	ns	0.7617
F2:Sample vs. F6:Erythromycin	****	<0.0001
F2:Sample vs. F6:Sample	****	<0.0001
F3:Erythromycin vs. F3:Sample	****	<0.0001
F3:Erythromycin vs. F4:Erythromycin	*	0.0174
F3:Erythromycin vs. F4:Sample	****	<0.0001
F3:Erythromycin vs. F5:Erythromycin	ns	0.7617
F3:Erythromycin vs. F5:Sample	****	<0.0001
F3:Erythromycin vs. F6:Erythromycin	****	<0.0001
F3:Erythromycin vs. F6:Sample	****	<0.0001
F3:Sample vs. F4:Erythromycin	****	<0.0001
F3:Sample vs. F4:Sample	****	<0.0001
F3:Sample vs. F5:Erythromycin	****	<0.0001
F3:Sample vs. F5:Sample	****	<0.0001
F3:Sample vs. F6:Erythromycin	*	0.0174
F3:Sample vs. F6:Sample	****	<0.0001
F4:Erythromycin vs. F4:Sample	****	<0.0001
F4:Erythromycin vs. F5:Erythromycin	****	<0.0001
F4:Erythromycin vs. F5:Sample	****	<0.0001
F4:Erythromycin vs. F6:Erythromycin	****	<0.0001
F4:Erythromycin vs. F6:Sample	****	<0.0001
F4:Sample vs. F5:Erythromycin	****	<0.0001
F4:Sample vs. F5:Sample	****	<0.0001

F4:Sample vs. F6:Erythromycin	****	<0.0001
F4:Sample vs. F6:Sample	*	0.0174
F5:Erythromycin vs. F5:Sample	*	0.0174
F5:Erythromycin vs. F6:Erythromycin	****	<0.0001
F5:Erythromycin vs. F6:Sample	****	<0.0001
F5:Sample vs. F6:Erythromycin	****	<0.0001
F5:Sample vs. F6:Sample	****	<0.0001
F6:Erythromycin vs. F6:Sample	****	<0.0001

For *E. coli*	Summary	Adjusted P Value
F1:Erythromycin vs. F1:Sample	****	<0.0001
F1:Erythromycin vs. F2:Erythromycin	**	0.0077
F1:Erythromycin vs. F2:Sample	****	<0.0001
F1:Erythromycin vs. F3:Erythromycin	**	0.0077
F1:Erythromycin vs. F3:Sample	****	<0.0001
F1:Erythromycin vs. F4:Erythromycin	ns	>0.9999
F1:Erythromycin vs. F4:Sample	****	<0.0001
F1:Erythromycin vs. F5:Erythromycin	ns	>0.9999
F1:Erythromycin vs. F5:Sample	****	<0.0001
F1:Erythromycin vs. F6:Erythromycin	**	0.0077
F1:Erythromycin vs. F6:Sample	****	<0.0001
F1:Sample vs. F2:Erythromycin	****	<0.0001
F1:Sample vs. F2:Sample	**	0.0077
F1:Sample vs. F3:Erythromycin	****	<0.0001
F1:Sample vs. F3:Sample	****	<0.0001
F1:Sample vs. F4:Erythromycin	****	<0.0001
F1:Sample vs. F4:Sample	**	0.0077
F1:Sample vs. F5:Erythromycin	****	<0.0001
F1:Sample vs. F5:Sample	****	<0.0001
F1:Sample vs. F6:Erythromycin	****	<0.0001
F1:Sample vs. F6:Sample	****	<0.0001

F2:Erythromycin vs. F2:Sample	****	<0.0001
F2:Erythromycin vs. F3:Erythromycin	ns	>0.9999
F2:Erythromycin vs. F3:Sample	****	<0.0001
F2:Erythromycin vs. F4:Erythromycin	**	0.0077
F2:Erythromycin vs. F4:Sample	****	<0.0001
F2:Erythromycin vs. F5:Erythromycin	**	0.0077
F2:Erythromycin vs. F5:Sample	****	<0.0001
F2:Erythromycin vs. F6:Erythromycin	ns	>0.9999
F2:Erythromycin vs. F6:Sample	****	<0.0001
F2:Sample vs. F3:Erythromycin	****	<0.0001
F2:Sample vs. F3:Sample	**	0.0077
F2:Sample vs. F4:Erythromycin	****	<0.0001
F2:Sample vs. F4:Sample	ns	>0.9999
F2:Sample vs. F5:Erythromycin	****	<0.0001
F2:Sample vs. F5:Sample	**	0.0077
F2:Sample vs. F6:Erythromycin	****	<0.0001
F2:Sample vs. F6:Sample	**	0.0077
F3:Erythromycin vs. F3:Sample	****	<0.0001
F3:Erythromycin vs. F4:Erythromycin	**	0.0077
F3:Erythromycin vs. F4:Sample	****	<0.0001
F3:Erythromycin vs. F5:Erythromycin	**	0.0077
F3:Erythromycin vs. F5:Sample	****	<0.0001
F3:Erythromycin vs. F6:Erythromycin	ns	>0.9999
F3:Erythromycin vs. F6:Sample	****	<0.0001
F3:Sample vs. F4:Erythromycin	****	<0.0001
F3:Sample vs. F4:Sample	**	0.0077
F3:Sample vs. F5:Erythromycin	****	<0.0001
F3:Sample vs. F5:Sample	ns	>0.9999
F3:Sample vs. F6:Erythromycin	****	<0.0001
F3:Sample vs. F6:Sample	ns	>0.9999
F4:Erythromycin vs. F4:Sample	****	<0.0001

F4:Erythromycin vs. F5:Erythromycin	ns	>0.9999
F4:Erythromycin vs. F5:Sample	****	<0.0001
F4:Erythromycin vs. F6:Erythromycin	**	0.0077
F4:Erythromycin vs. F6:Sample	****	<0.0001
F4:Sample vs. F5:Erythromycin	****	<0.0001
F4:Sample vs. F5:Sample	**	0.0077
F4:Sample vs. F6:Erythromycin	****	<0.0001
F4:Sample vs. F6:Sample	**	0.0077
F5:Erythromycin vs. F5:Sample	****	<0.0001
F5:Erythromycin vs. F6:Erythromycin	**	0,0077
F5:Erythromycin vs. F6:Sample	****	<0.0001
F5:Sample vs. F6:Erythromycin	****	<0.0001
F5:Sample vs. F6:Sample	ns	>0.9999
F6:Erythromycin vs. F6:Sample	****	<0.0001

3. RESULT AND DISCUSION

3.1.1 Contact Angle Study

The contact angle images are consistent with the obtained measurements. According to these images, the lowest contact angle—0 degrees—was observed in the F6 formulation. While the incorporation of HA in the F2 formulation led to a reduction in contact angle, the addition of *Cotinus coggygria* extract resulted in a markedly more significant decrease.

3.1.2 Mechanical Properties

Analysis of the stress–strain curves revealed that the highest tensile stress was observed in the F3 formulation, which was prepared with PCL and 1% *Cotinus coggygria* extract. A comparable tensile stress value was recorded for the F4 formulation, in which the extract concentration was increased to 3%. Notably, the highest tensile strain was also observed in the F4 formulation. Although the F5 formulation exhibited lower tensile stress and strain values compared to F3 and F4, it demonstrated the highest tensile strength. Overall, the addition of HA to the extract-containing formulations resulted in a marked decrease in both tensile stress and tensile strain [1].

REFERENCE

[1] Esfanjani, E., Ramezani, A., Sanjarian, F., & Shafiei, S. S. Electrospun polycaprolactone coated with gum tragacanth containing layered double hydroxide/thymol nanohybrid for wound dressing application. International Journal of Biological Macromolecules, 2025, 143049. DOI: 10.1016/j.ijbiomac.2025.143049

Specifications of the extracts provided by VEMO.

VEMO 99 Ltd
Address: 88 Rozhen Blvd., 1271 Sofia, Bulgaria
Tel./Fax: +359 2 931 10 91 | Tel.: +359 2 931 66 21
E-mail: info@vemo-vsv.com | Web: www.vemo-vsv.com

VEMO PREMIUM QUALITY FOR ALL

Specification

Product Name:	**Cotinus coggygria** dry extract
Name of the plant:	Cotinus coggygria
Part of the plant used:	Folia

Characteristics and standards

1.	Appearance	Amorphous powder
2.	Colour	Light brown to brown
3.	Taste & Odour	Specific to plants
4.	Loss on drying, %, not more than	5.0
5.	Total polyphenols, determined as catechin, based on dry substance, %, not less than	30.0
6.	Tannic compounds, determined as tannin, based on dry substance, %, not less than	32.0
7.	Flavonoids, based on dry substance, %, not less than, determined as:	
	- apigenin	15.0
	- quercetin	2.0
8.	Heavy metals, mg/kg, not more than:	
	- Lead (Pb)	3.0
	- Cadmium (Cd)	1.0
	- Mercury (Hg)	0.1
9.	Microbiology	
	Total Viable count, CFU/g, not more than	1.10^4
	Mould and Yeast, CFU/g, not more than	1.10^3
	E.coli, Salmonella spp., S. aureus, CFU/g	Must not be detected
10.	Content of extraction solvent residues	Must comply with Ph.Eur.
11.	Excipients	None
12.	Packaging	2 Polyethylene bags placed in a five layer carton box: 10.0 kg net per box
13.	Storage	Store in dry and cool places, away from heat and direct sunlight
14.	Shelf life	36 months under the previously mentioned conditions in its original packaging

Manufactured and controlled according to the principles and requirements of
EN ISO 9001-2015, HACCP, FSSC 22000:2011, GMP, GMP+, Halal, Kosher

VEMOHERB
HERBAL EXTRACTS

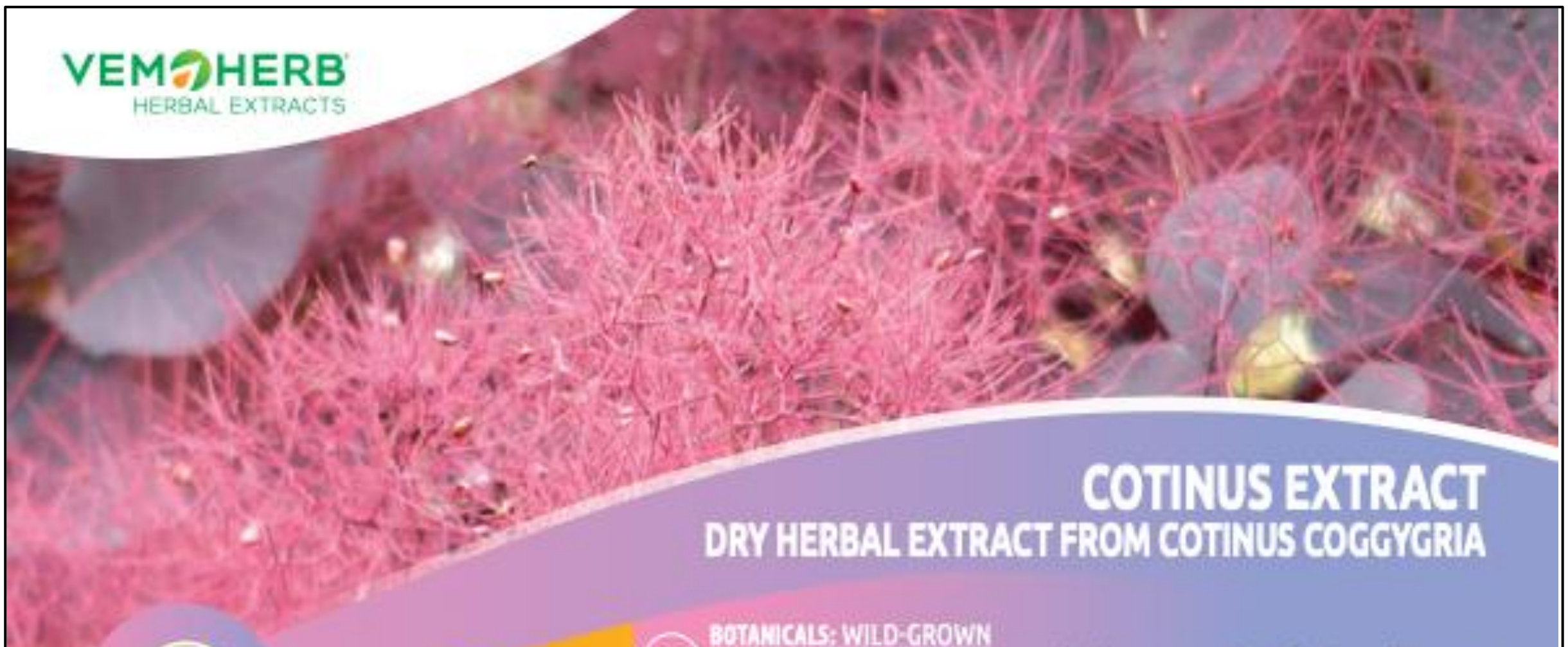

COTINUS EXTRACT

DRY HERBAL EXTRACT FROM COTINUS COGGYGRIA

BOTANICALS: WILD-GROWN
Wild-grown herbs are stronger and with greater variety of active substances, when compared to the cultivated ones, providing for their actual benefits.

ORIGIN: BULGARIAN
Based in southeastern EU, famous for its great diversity of ecologically clean botanicals.

PURITY: FLAWLESS
VemoHerb® extracts are completely clean from unwanted ingredients and are concentrated to maximum levels of active substances.

QUALITY: EXCELLENT
Complete quality control of each batch is performed, providing for the constant properties of our products.

Wild Grown

CHARACTERISTICS AND STANDARDS

Appearance	Amorphous powder
Colour	Light-Brown to Brown
Taste & Odour	Specific to plants
Loss on drying, %, not more than	5.0
Heavy metals, mg/kg, not more than	
• Lead (Pb)	3.0
• Cadmium (Cd)	1.0
• Mercury (Hg)	0.1
Tannic compounds, determined as tannin, based on dry substance, %, not less than	32.0
Total polyphenols, determined as catechin, based on dry substance, %, not less than	30.0
Flavonoids, based on dry substance, %, not less than, determined as:	
• apigenin	15.0
• quercetin	2.0
Microbiology	
• Total viable count, CFU/g, not more than	1.10^4
• Mould and Yeast, CFU/g, not more than	1.10^3
• E. coli, Salmonella spp., S. aureus, CFU/g	Must not be detected
Content of extraction solvent residues	Must comply with Ph.Eur.
Excipients	None
Packaging	2 polyethylene bags placed in a five-layer carton box: 10.0 kg net per box
Storage	Store in dry and cool places, away from heat and direct sunlight
Shelf life	36 months in its original packaging

Manufactured and controlled according to the principles and requirements of EN ISO 9001-2015, HACCP, FSSC 22000, GMP, GMP+, Halal, Kosher

THE PLANT

Cotinus coggygria, also known as smoke tree or smoke bush, is an interesting large bush with multiple bioactivities. The plant can be found in the southern mountain slopes on dry and stony soils. Being one of the 15 most popular plants in the Bulgarian folk medicine, it is used for different wounds and cases of acne as an external means, as it is believed to show antimicrobial and hepatoprotective properties.

THE MANUFACTURING PROCESS

Our unique know-how technology has been developed to perfection throughout the last 20 years. The manufacturing process employed in the production guarantees complete extraction of the active substances present in the used parts of the herb.

BIOLOGICALLY ACTIVE SUBSTANCES

The unquestionable properties of the dry extract from *Cotinus coggygria* are due to the content of specific active ingredients extracted from the plant, containing:

- polyphenols, namely catechin,
- the flavonoids apigenin and quercetin,
- and tannins, among other active substances present.

MODE OF ACTION AND APPLICATIONS

In the folk medicine, a brew of *Cotinus coggygria* is used externally in cases of wounds and catarrhs of various origin, and internally for supporting the mucosal health. The main active substances in the plant are polyphenols, tannins and flavonoids. They are heterogeneous chemical compound group that generally induces protein coagulation by binding irreversible to them. This property is observed in the astringent effects of the products based on the plant. Additionally, the active compounds present in the plant show the ability to increase the resorption of certain vitamins in the body and help maintain the healthy cholesterol levels in the blood. Also, the *Cotinus coggygria* extract was shown to have a beneficial effect when administered as dermatological agent, enhancing the elasticity and structural integrity of the skin.

VEMO | PREMIUM QUALITY FOR ALL

VEMO 99 Ltd – 88 Rozhen Blvd., 1271 Sofia, Bulgaria
phone: +359 2 931 66 21, e-mail: info@vemo-vsv.com
www.vemo-vsv.com; www.vemoherb.com

VEMO 99 Ltd
Address: 88 Rozhen Blvd., 1271 Sofia, Bulgaria
Tel./Fax: +359 2 931 10 91 | Tel.: +359 2 931 66 21
E-mail: info@vemo-vsv.com | Web: www.vemo-vsv.com

Analytical method of dry extract from *Cotinus coggygria*

I. General characteristics.

1. Appearance and colour: visually estimated.

2. Odour: Organoleptic estimation.

3. Water: The testing is made in correspondence with Ph. Eur 2.2.32. "Loss in Drying"

0.500 product is dried at 105°C up to permanent weight.

4. Heavy metals: In correspondence with Ph. Eur, 2.4.8. "Heavy metals" and BS EN 14082:2003

5. Microbiological parameters: The testing is made in correspondence with Ph. Eur. 2.6.12. "Total viable aerobic count", Ph. Eur. 2.6.13. "Test for specified micro-organisms", BS EN ISO 4833-1:2013, BS ISO 21527-2:2011, ISO 16649-2:2001, BSS EN ISO 6579:2003 and BSS EN ISO 6888-1:2005.

II. Active Ingredients.

1. **Content of polyphenols, estimated as catechin**

1. Definition

The testing is made in correspondence with Ph. Eur. 2.2.25. "Absorption Spectrophotometry in Ultraviolet and Visible Area"

Solvent: 70 % methanol.

Reagent: Folin-Ciocalteo, reagent of Merck

Test solution: To 25,0 mg substance are added 40 ml Solvent and the solution is heated at temperature of 40oC for 30 minutes and stirred periodically. It is cooled, filled up to 50.0 ml with Solvent and is filtered through a paper filter 'blue band', and the first filtrate portions are thrown away.

To 0,5 ml filtrate are added 0,5 ml Reagent, after homogenization are added 10 ml solution of sodium carbonate (7.5 %). The solution is homogenized again and the volume is made up to 25.0 ml with water. The solution is kept at room temperature for 60 min. After 60 min is filtered through a paper filter 'blue band'.

Standard solution: 10,0 mg catechin reference substance, reagent of Fluka are dissolved to 50,0 ml with Solvent.

0,5 ml from Standard solution are developed as Test solution.

Control solution: 0.5 ml methanol (70%) are developed as Test solution.

VEMO 99 Ltd
Address: 88 Rozhen Blvd., 1271 Sofia, Bulgaria
Tel./Fax: +359 2 931 10 91 | Tel.: +359 2 931 66 21
E-mail: info@vemo-vsv.com | Web: www.vemo-vsv.com

The absorptions of the Test solution and Standard solution are measured at wavelength of 725 nm in relation to Control solution.

The content of polyphenols, estimated as catechin in the product, is calculated from the measured absorption and the concentration of Test solution and Standard solution, and the concentration of the solutions

$$\% \text{ total polyphenols} = \frac{\Delta A}{A\ catechin} * \frac{0.010}{50} * \frac{50}{G} * 100$$

2. **Content of tannic compounds, estimated as tannin**

It is accomplished in agreement with DF X.

80 ml water is added to 0.200g of the tested substance and is heated in boiling water-bath for 30 minutes, with a concomitant frequent stir. After cooling, the solution is filled up to 100.0 ml of water and is filtered with an ordinary paper filter, and the first filtrate portions were thrown away.

100.0 ml water and 5.0 ml reagent solution are added to 50.0 ml of the filtrate and the resulting solution is titrated with 0.02 mol/l potassium permanganate – KMnO4 untill yellow colouration

Control solution: 5.0 ml Reagent are titrated under the same conditions as the Test solution.

1 ml solution of potassium permanganate - KmnO4 (0.02 mol/l) equates to 4.157 mg tannic compounds, estimated as tannin.

Reagent: 1.0 g indigo carmine ($C_{16}H_8N_2Na_2O_8S_6$) is dissolved in 6% sulphuric acid up to a total volume of 1000.0 ml

$$\frac{(V-Vo)\ .\ 100.\ 0.004157}{G\ .\ V(sample)}\ .100\ \%$$

V – volume of potassium permanganate, used to titrate the sample

Vo - volume of potassium permanganate, used to titrate the control

100 – pre-dilution of the sample

0.004157 – coefficient, corresponding to 1 ml of potassium permanganate

G – sample mass

V(sample) – volume of the sample, used for the titration

3. Content of flavonoids, estimated as apigenin and quercetin

The testing is made in correspondence with Ph.Eur. 2.2.25. "Absorption Spectrophotometry in Ultraviolet and Visible Area"

Solution I: 10.0 g aluminium chloride (hexahydrate) are dissolved in 10.0 ml of water and the solution is reduced to 100.0 ml with ethanol (96%).

NOTE: If necessary, before dilution, the water solution of aluminium chloride can be heated in a water-bath to induce complete dissolving.

Solution II: 10.0 g ammonium acetate are dissolved in 40.0 ml of water. pH of the solution is measured and the value is corrected to pH 6.0 with ammonia (250 g/l), or with acetic acid (60 g/l). After correction of pH of the solution, the acetate buffer is reduced to 50.0 ml with water.

Test solution: To 25.00 mg of product are added 40 ml ethanol (96%) and the obtained solution is shaken at a temperature 30 – 35 °C for 30 min. After cooling, the solution is filled up to 50.0 ml with ethanol (96%) and is filtered through a paper filter "blue band", and the first portions of filtrate are thrown away.

To 4.0 ml of filtrate is added 1.0 ml *Solution I,* 1.0 ml *Solution II* and it is filled up to 10.0 ml with ethanol (96%).

Standard solution I: 5.0 mg apigenin, referent substance (Merck reagent) are dissolved up to 100.0 ml with ethanol (96%).

1.0 ml of the obtained solution is developed as *Test solution*.

Standard solution II: 3.0 mg quercetin, referent substance (Merck reagent) are dissolved up to 100.0 ml with ethanol (96%).

2.0 ml of the obtained solution is developed as *Test solution*.

Control solution: 1.0 ml ethanol (96%) is developed as *Test solution.*

Address: 88 Rozhen Blvd., 1271 Sofia, Bulgaria
Tel./Fax: +359 2 931 10 91 | Tel.: +359 2 931 66 21
E-mail: info@vemo-vsv.com | Web: www.vemo-vsv.com

The absorptions of the *Test solution*, *Standard solution I* and *Standard solution II* are measured at wavelength of 420 nm and layer thickness of 1 cm, in relation to *Control solution*.

The content of flavonoids in the product, estimated as apigenin and quercetin, is determined from the reported values of solutions absorption and concentration.
